# Differentiating Plasmon-Enhanced Chemical Reactions on AgPd Hollow Nanoplates through Surface-Enhanced Raman Spectroscopy

**DOI:** 10.1021/acscatal.3c06253

**Published:** 2024-04-17

**Authors:** Sulin Jiao, Kun Dai, Lucas V. Besteiro, Hongen Gao, Xuan Chen, Weichao Wang, Yuan Zhang, Chuntai Liu, Ignacio Pérez-Juste, Jorge Pérez-Juste, Isabel Pastoriza-Santos, Guangchao Zheng

**Affiliations:** † Key Laboratory of Materials Physics, Ministry of Education, School of Physics, Zhengzhou University, Zhengzhou 450001, P. R. China; ‡ School of Materials Science and Engineering, Key Laboratory of Materials Processing and Mold, Zhengzhou University, Zhengzhou 450001, P. R. China; § CINBIO, Universidade de Vigo, Campus Universitario As Lagoas, Marcosende, 36310 Vigo, Spain; ∥ Departamento de Física Aplicada, Universidade de Vigo, Campus Universitario As Lagoas, Marcosende, 36310 Vigo, Spain; ⊥ Departamento de Química Física, Universidade de Vigo, Campus Universitario As Lagoas, Marcosende, 36310 Vigo, Spain; # Institute of Quantum Materials and Physics, Henan Academy of Sciences, Zhengzhou 450046, China

**Keywords:** AgPd nanoplates, hot electrons, Joule effect, photocatalysis, in situ SERS

## Abstract

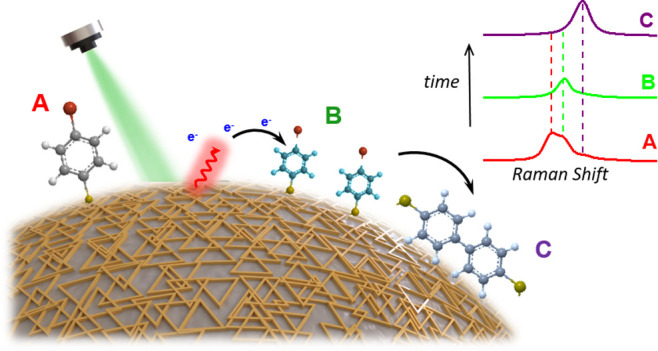

Plasmonic photocatalysis demonstrates great potential for efficiently harnessing light energy. However, the underlying mechanisms remain enigmatic due to the transient nature of the reaction processes. Typically, plasmonic photocatalysis relies on the excitation of surface plasmon resonance (SPR) in plasmonic materials, such as metal nanoparticles, leading to the generation of high-energy or “hot electrons”, albeit accompanied by photothermal heating or Joule effect. The ability of hot electrons to participate in chemical reactions is one of the key mechanisms, underlying the enhanced photocatalytic activity observed in plasmonic photocatalysis. Interestingly, surface-enhanced Raman scattering (SERS) spectroscopy allows the analysis of chemical reactions driven by hot electrons, as both SERS and hot electrons stem from the decay of SPR and occur at the hot spots. Herein, we propose a highly efficient SERS substrate based on cellulose paper loaded with either Ag nanoplates (Ag NPs) or AgPd hollow nanoplates (AgPd HNPs) for the in situ monitoring of C–C homocoupling reactions. The data analysis allowed us to disentangle the impact of hot electrons and the Joule effect on plasmon-enhanced photocatalysis. Computational simulations revealed an increase in the rate of excitation of hot carriers from single/isolated AgPd HNPs to an in-plane with a vertical stacking assembly, suggesting its promise as a photocatalyst under broadband light. In addition, the results suggest that the incorporation of Pd into an alloy with plasmonic properties may enhance its catalytic performance under light irradiation due to the collection of plasmon-excitation-induced hot electrons. This work has demonstrated the performance-oriented synthesis of hybrid nanostructures, providing a unique route to uncover the mechanism of plasmon-enhanced photocatalysis.

Nanoplasmonics is a developing area of research that investigates the interaction between light and matter at the nanometer scale through resonant excitations of surface plasmons in metallic nanostructures called localized surface plasmon resonance (LSPR).^1−3^ Upon plasmon excitation, a strong electric field is confined at the surface of the nanoparticle and the plasmon decays via radiative and nonradiative pathways. In the nonradiative pathway, the plasmon decay generates an energetic electron–hole pair within the metal, often referred to as hot electrons and hot holes (hot carriers), with a nonthermal distribution that eventually relaxes through electron–electron scattering and electron–phonon interaction, generating heat (Joule effect). Several LSPR-based applications have been developed by exploiting nonradiative decay in the fields of sensing, catalysis, and electronics.^4−6^

In the context of plasmonic photocatalysis, the hot carriers may be either deposited into the acceptor states of adsorbates or transferred to an accepting medium, creating charge-separated states with enough chemical potential energy to facilitate chemical (redox) reactions.^7−11^ Moreover, in addition to the appropriate energy, the photocatalytic efficiency may also be hindered by the short lifetimes and mean free path of hot carriers. Recently, researchers have endeavored to enhance charge separation at the interfaces of metals and other nanodomains, including semiconductors, with the aim of extending the lifetime of energetic electrons.^12,13^ Alternatively, in a chemically inert environment, hot carriers may transfer their energy to the metal lattice, resulting in nanoparticle heating. This heating could eventually lead to the transfer of energy to adsorbates, driving chemical transformations through an Arrhenius dependence of the reaction rate on surface temperature.^14^ Recently, Baffou et al.^15^ reviewed different simple experimental procedures reported in the literature for detecting and quantifying photothermal effects and for discriminating their contribution from that due to photochemical processes in plasmon-driven chemical reactions. For instance, varying the illumination power can be utilized to estimate the relative contributions of photothermal and photochemical effects. However, this approach requires a significant range of power variation, often leading to considerable changes in the reaction rate over several orders of magnitude.^16^ Alternatively, variations in rate enhancement at different wavelengths do not necessarily imply a plasmonic hot-carrier-driven process, as near-field enhancements may not always directly correlate with sample absorbance. Nevertheless, such variations do provide compelling evidence for the presence of a photochemical process.^17^ The fundamentals of photothermal heating rely on the slow removal of deposited heat from the nonradiative decay. Consequently, heat accumulates in the irradiated volume, resulting in a localized temperature rise. Interestingly, when nanoparticles are well-dispersed in a liquid, the interaction between individual particles and the liquid medium facilitates heat transfer by conduction, enabling effective heat dissipation. Furthermore, stirring the ensemble further enhances both heat and mass transport, ensuring the homogenization of heat.^18^

Various model reactions and techniques have been suggested to disentangle the behavior of hot electrons in a plasmon-induced chemical reaction.^19^ A particularly intriguing approach involves the use of surface-enhanced Raman scattering (SERS) spectroscopy to characterize chemical reactions driven by hot electrons.^10,20,21^ This is noteworthy, as both SERS and hot electrons originate from the decay of LSPR and occur within the hot spot. Recently, using the reduction of *para*-aminothiophenol (*p*-ATP) to 4,4′-dimercaptoazobenzene (DMAB) as the model reaction, the hot electron transfer at different interfaces (plasmonic metal–molecule, plasmonic metal–metal, plasmonic metal–semiconductor, and plasmonic metal–insulator) and the role of hot electrons in the photochemical process were explored via in situ SERS.^10,20,21^ Additionally, the reduction of methylene blue (MB) has been proposed as a model system to demonstrate the direct transfer mechanism of the hot electrons into the antibonding orbital of MB to initiate chemical reactions.^22^

An optimal catalytic platform for in situ SERS analysis should exhibit high SERS performance, catalytic activity, and excellent stability.^23−25^ This ensures that the reactant, intermediates and products can be readily discerned through a time-resolved SERS study.^26^ Herein, we propose a dip catalyst based on filter paper loaded with plasmonic nanoparticles for real-time SERS monitoring of a C–C bond formation (Ullmann type reaction), serving as a model reaction to distinguish plasmon-enhanced chemical reactions.^27^ Particularly, we have chosen Ag nanoplates (NPs) and AgPd hollow nanoplates (HNPs) as plasmonic enhancers to investigate the efficiency of hot electron generation along with the synergistic effect of Pd in promoting C–C bond formation reactions. Ag NPs were synthesized using a previously reported seed-mediated approach,^13^ while AgPd HNPs were synthesized through a template-assisted approach (for detailed information, see the Experimental Section in the Supporting Information (SI)).^28^Figure 1a shows the extinction spectrum of Ag NPs with three well-defined LSPR bands located at 586, 412, and 338 nm attributed to the in-plane dipole mode, the out-of-plane dipole mode, and the out-of-plane quadrupole mode, respectively. Figure S1 in the SI provides a detailed transmission electron microscopy (TEM) and high-resolution TEM (HRTEM) characterization of as-prepared Ag NPs and the resulting size distribution histograms (length: 52.8 ± 5.2 nm; thickness: ca. 14 nm). These Ag NPs are subsequently employed as sacrificial templates for the synthesis of AgPd HNPs through a controlled galvanic replacement reaction, achieved by the addition of sodium tetrachloropalladate as the Pd precursor (for detailed information, refer to the Experimental Section in the SI). The galvanic replacement process was monitored by time-resolved ultraviolet–visible–near-infrared (UV–vis–NIR) spectroscopy (Figure S2), revealing the disappearance of the low-energy modes, while the in-plane dipole plasmon resonance was red-shifted from 586 to 1044 nm and dampened (Figure 1a). In this process, Pd atoms are epitaxially deposited onto the edges of Ag templates, specifically the {110} facets, which have the highest surface energy (γ{111} < γ{100} < γ{110}).^28,29^Figures 1b,c and S2 in the SI display representative TEM and scanning electron microscopy (SEM) images of the obtained nanoparticles, revealing their nanoring morphology. These nanostructures maintain the initial shape of Ag NPs and possess an inner diameter of 57.6 ± 8.4 nm and a nanoring thickness of 13.8 ± 3.1 nm (Figure S2 in the SI). Additionally, high-magnification selected area electron diffraction (SAED) and energy-dispersive X-ray spectroscopy mapping unveil the polycrystalline nature of the nanorings and the homogeneous distribution of both Ag and Pd, with an average Ag/Pd ratio of 9.8:98.2 (Figure 1e). HRTEM analysis clearly indicates the presence of lattice fringes with 0.24 and 0.22 nm spacing assigned to the Ag(111) and Pd(111) planes, respectively (Figure 1d). X-ray diffraction (XRD) pattern (Figure S3 in the SI) shows peaks corresponding to a face-centered cubic (fcc) crystal structure.

**Figure 1:**
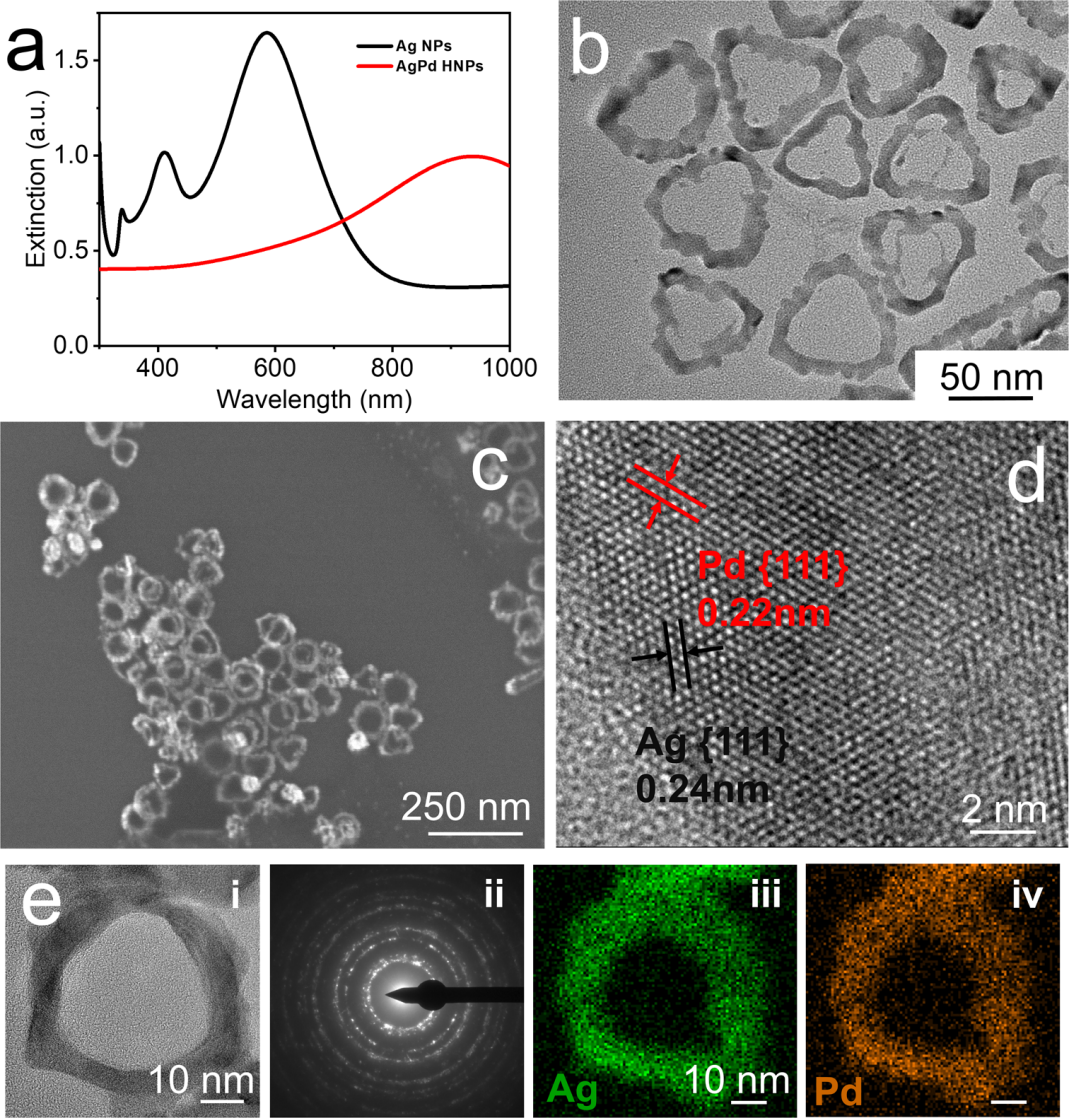
(a) UV–vis–NIR extinction spectra of Ag nanoplates (black line) and AgPd nanorings (red line). (b, c) Representative TEM and SEM images of the AgPd nanorings, respectively. (d) HRTEM image of a AgPd nanoring indicating the presence of lattice fringes with 0.24 and 0.22 nm spacing assigned to the Ag(111) and Pd(111) planes, respectively. (e) HRTEM image of a AgPd nanoring (i), the corresponding SAED pattern (ii), and the EDX mapping analysis for the distribution of Ag (iii) and Pd (iv).

Colloidal stability is crucial for the in situ SERS monitoring of chemical reaction processes on the nanocatalyst surface. Therefore, assembling the nanoparticles on a solid support not only ensures their stability but also provides a high concentration of randomly distributed hot spots.^30,31^ In this work, we chose a dip catalyst based on cellulose paper.^32,33^ Thus, the paper strip (typically 1.0 cm width and 1.0 cm length, Figure 2a) was immersed in the Ag NP or AgPd HNP dispersions for an appropriate duration. Subsequently, the paper strip was withdrawn and dried at 60 °C. To maximize nanoparticle loading, the dipping process was repeated up to 6 times, observing an increase in color intensity with each deposition step when assessed by the naked eye (Figure S4 in the SI). The nanocomposites were characterized by SEM, showing that both the Ag and AgPd HNPs are densely packed and adsorbed onto the surface of cellulose fibers (Figure 2a), so they are expected to produce intense SERS signals. It should be pointed out that no desorption of nanoparticles from the paper was observed after drying, which confirmed a tight adsorption of the nanoparticles to the substrate. The loading process relies on electrostatic interactions between the positively charged CTAB-stabilized Ag or AgPd HNPs and the negatively charged cellulose fibers due to the presence of carboxyl, sulfonic acid, phenolic, or hydroxyl groups.^34^ Interestingly, the optical properties of the AgPd HNP-doped filter paper show a strong plasmonic coupling, as indicated by the broad band in reflectance (Figure S4e in the SI).

**Figure 2:**
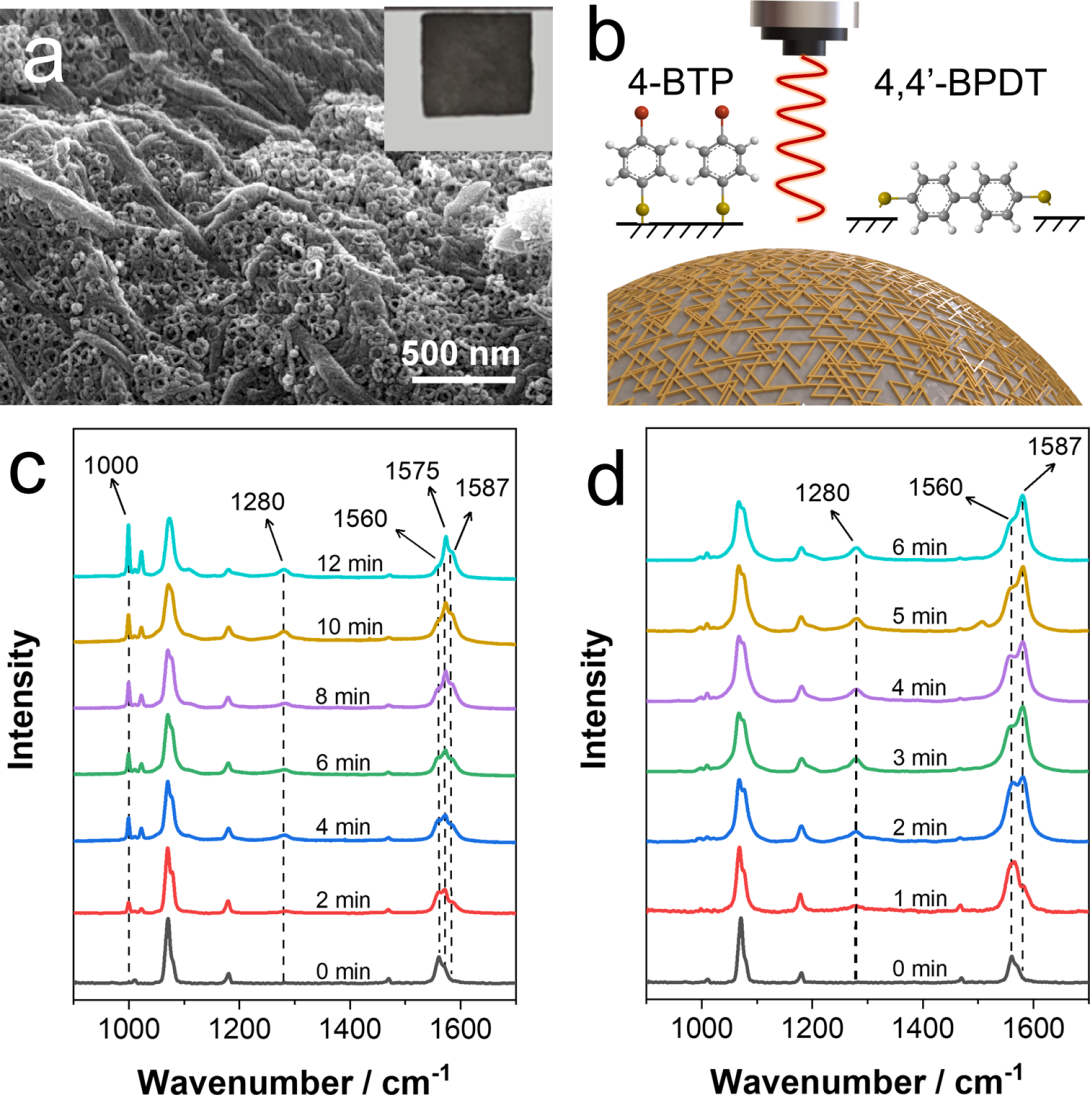
(a) Representative SEM image of the AgPd HNP-loaded filter paper. The inset shows an image of a 1 × 1 cm^2^ AgPd HNP-loaded filter paper. (b) Schematic representation of the photocatalyzed C–C homocoupling reaction. (c, d) Time-resolved SERS spectra of the C–C homocoupling reaction on the Ag NP-loaded (c) and AgPd HNP-loaded substrate. SERS measurements were carried out with a 633 nm laser line, 50× objective, 0.4 mW/cm^2^ laser power, and an acquisition time of 15 s.

Next, we studied the catalytic efficiency of both substrates, Ag NP- and AgPd HNP-loaded filter paper, toward the C–C homocoupling reaction of 4-bromothiophenol (4-BTP) to 4,4′-biphenyldithiol (4,4′-BPDT) upon light irradiation with a 633 nm laser line and basic conditions. Before starting the experiment, 4-BTP molecules were attached on the nanoparticle surface using thiol chemistry. The presence of 4-BTP on the Ag NP- and AgPd HNP-loaded filter paper was confirmed by SERS. As shown in Figure 2c,2d (time 0) for Ag NP or AgPd HNP substrate, respectively, the SERS spectra recorded present two bands located at 1079 cm^–1^ (to ring deformation) and 1560 cm^–1^ (C=C symmetric stretching) of 4-BTP. A complete SERS assignment is displayed in Figure S5 and Table S1 in the SI. Upon 633 nm laser line irradiation, the time evolution SERS spectra clearly indicate that two bands located at 1280 and 1587 cm^–1^, attributed to 4,4′-BPDT, arise. In addition, two low-intensity bands at 1000 and 1575 cm^–1^, attributed to 4-thiophenol (4-TP), also appear (see Figure S5 in the SI for a full assignment of 4-TP and 4,4′-BPDT). It should be noted that the relative intensity of these bands varies as a function of the SERS substrate. Therefore, throughout the reaction, the measured SERS spectra could be attributed to different percentages of the reactant (4-BTP) and the products (4-TP and 4,4′-BPDT). Interestingly, since 4-BTP, 4-TP, and 4,4′-BPDT can be easily differentiated by SERS (Figure S6 in the SI), it is possible to obtain quantitative information on the relative concentrations of the three molecules. This is achieved by correcting their characteristic peak areas according to their SERS cross sections, as determined by SERS measurements under identical experimental conditions (Figure S7a,b). Figure S7c displays the relative concentration of the reaction products 4,4′-BPDT and TP as determined by SERS analysis. The time-dependent analysis indicates that the homocoupling reaction occurs in both SERS substrates. However, the relative concentration of 4,4′-BPDT is higher in the AgPd HNP substrate, and the formation of the dehalogenated intermediate 4-TP is predominant in the Ag NP substrate, as demonstrated by its prominent bands at 1000 and 1575 cm^–1^ (Figure 2).

Considering the higher photocatalytic efficiency of the AgPd HNP-loaded filter paper toward the homocoupling reaction, we attempted to disentangle the hot electron and photothermal heating effects by analyzing the influence of the laser power, laser wavelength, and temperature. Figure S8 illustrates the influence of the 633 nm laser power on the time-resolved SERS analysis of the reaction. This data was used to estimate the relative concentration of 4,4′-BPDT produced along the reaction as a function of the laser power (Figure 3a). Notably, the relative concentration of 4,4′-BPDT produced increases along with the extent of the reaction, as the laser power density rises. Interestingly, an increase in the 633 nm laser power from 0.1 to 0.4 mW/cm^2^ led to an increase in the relative concentration of 4,4′-BPDT produced from 30 to 52%, suggesting a hot electron driven behavior. No attempt was made to estimate the hot-electron-driven behavior based on conversion rates of 4,4′-BPDT, as two reaction pathways are involved, and this influences the final 4,4′-BPDT conversion. Similar results were obtained for the 532 nm laser line (Figure S9, SI). These results suggest a correlation between the reaction and illumination power, implying that the LSPR excitation drives the reaction. In order to rule out the photothermal effect, we performed the reaction in a closed temperature control device, maintaining the substrates at a stable temperature during the in situ SERS analysis (Figure S10). Figure 3b shows the relative 4,4′-BPDT concentration as a function of the temperature (298.15, 313.15, and 353.15 K). The results demonstrate that both the extent and rate of product formation remain constant within the studied temperature range. Furthermore, these findings may eliminate the possibility that the reaction is triggered by brief yet exceptionally high local temperatures at the surface of the nanoparticles prior to attaining a thermal steady state. Given that we are utilizing continuous wave laser illumination and our data indicates the reaction advancing over the course of minutes, a time frame consistent with a steady-state scenario, it becomes evident that highly nonequilibrium states with subnanosecond lifespans are unlikely contributors.

**Figure 3:**
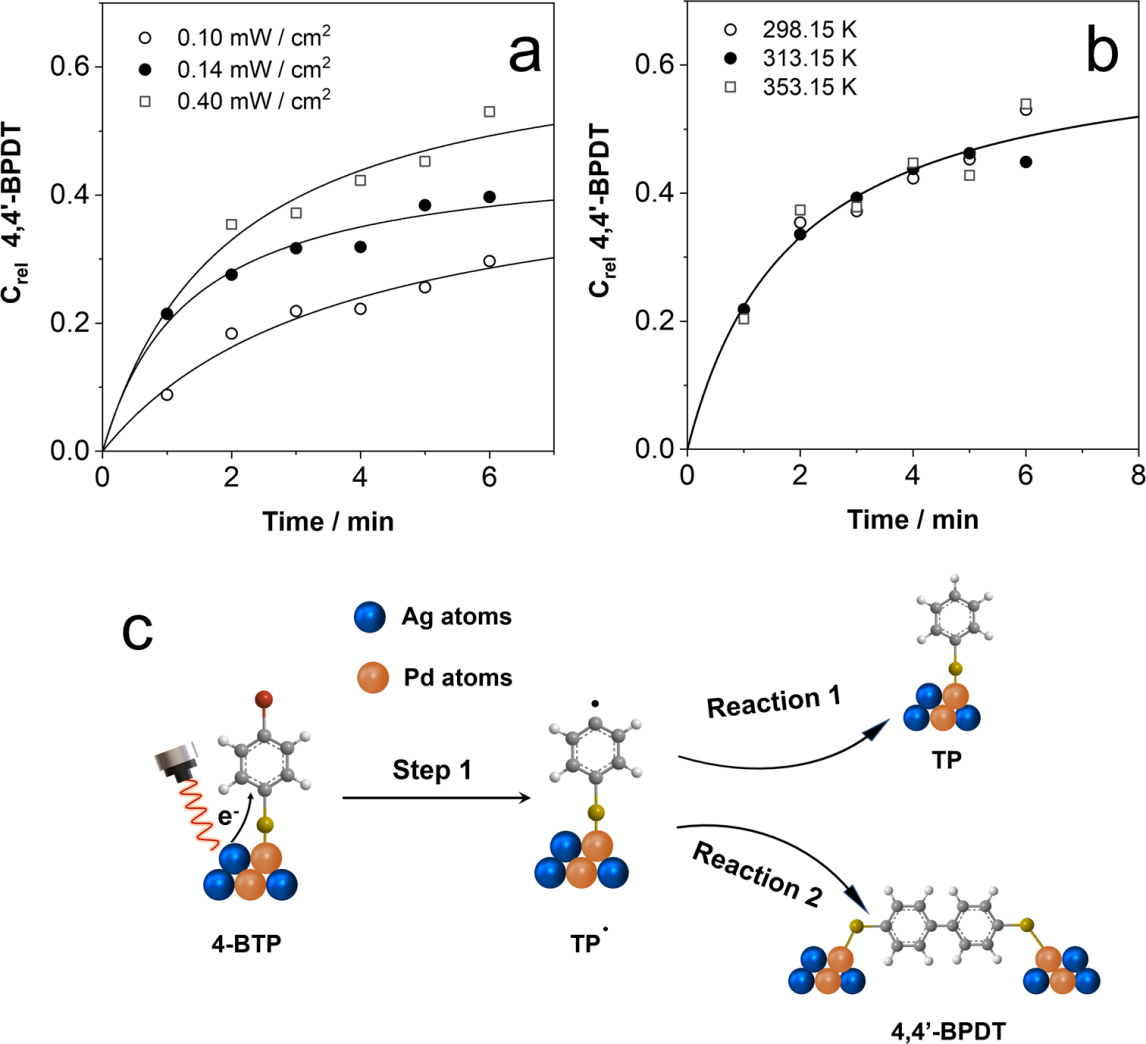
(a, b) Relative 4,4′-BPDT concentration estimated during the homocoupling reaction via in situ SERS measurements on a AgPd HNP-loaded filter paper substrate under 633 nm laser excitation at different power densities (a), as indicated, and different temperatures (b) with a power density of 0.4 mW/cm^2^. All spectra were recorded with a 50× objective and 15 s acquisition time. The lines are a guide to the eye. (c) Schematic of the proposed mechanism with two possible pathways.

To summarize, the results confirm that upon light irradiation, the main product on the Ag NP substrate is the dehalogenation of 4-BTP (4-TP), whereas on the AgPd HNP substrate, the main product is the homocoupling (4,4′-BPDT). Interestingly, in both cases, the reaction yields are higher under basic conditions. Thus, Figure S11 (SI) shows the dehalogenation of 4-BTP in the presence and absence of NaOH (pH 14) on the Ag NP substrate. A higher 4-TP ratio is observed at pH 14, as demonstrated by the enhanced intensity of the characteristic SERS peaks of thiophenol at 1000 and 1575 cm^–1^. Such behavior could be explained by the generation of ^•^OH radicals in the reaction medium through the oxidation of hydroxide ions on the photocatalyst surface by photogenerated holes.^35^ These radicals act as hole sacrificial reagents, allowing for the continuous generation of hot electrons.

In addition, for the homocoupling reaction, the extent is higher at higher pH because the basic medium facilitates the required deprotonation.^36^ Photoinduced homocoupling C–C bond formation has been proposed to proceed through a radical mechanism,^27^ involving the reduction of 4-BTP to its radical anion, which then dissociates into TP· and Br^–^. Eventually, TP can be reduced by hot electrons and subsequently combined with H^+^ in solution to form TP (pathway 1 in Figure 3), or two adjacent TP^•^ radicals may undergo a self-coupling reaction to form 4,4′-BPDT (pathway 2 in Figure 3). Besides, hot electrons generated upon laser illumination cannot break the C–H bond since the bond energy of C–H (413 kJ/mol) is much higher than that of C–Br (276 kJ/mol).^37^ This is demonstrated by the absence of the characteristic SERS peaks of 4,4′-BPDT when a monolayer of TP molecules on a AgPd HNP substrate is irradiated with a 633 nm laser line (Figure S12).

Furthermore, it is known that C–C coupling reactions can also be catalyzed heterogeneously on supported Pd NPs, but all of them require moderate to elevated temperatures (>60 °C).^38^ Interestingly, incorporating Pd into an alloy with plasmonic properties may enhance the catalytic performance of palladium under visible laser irradiation and also at room temperature due to the collection of LSPR-induced hot electrons. This results in energetic electrons collecting at the Pd sites on the NP surface. Therefore, it is reasonable to expect that under visible irradiation, the Pd sites with the energetic electrons at the alloy NP surface will exhibit superior catalytic activity compared to pure Pd NPs alone, even at room temperature. These findings indicate that the AgPd alloy NPs function as photocatalysts, significantly enhancing the catalytic performance of Pd for organic reactions under visible light irradiation at ambient temperatures. In addition, the AgPd hollow nanostructures exhibit not only high surface area but also enhance the strong light–matter absorption through LSPR behavior and multiple photon scattering.^39^

Next, we performed a series of computational studies to explore some aspects of the photocatalytic response of the AgPd HNPs. This investigation encompassed their behavior as independent particles and, importantly, to connect with the complex NP distribution on the paper substrate (Figure 2a), in interparticle interaction with their neighbors. Our analysis uses a previously discussed theoretical model for computing the excitation rates of intraband hot carriers.^40−42^ After constructing computational models for alloyed AgPd HNP with optical properties similar to those of the experimental AgPd HNPs (Figure S13), we studied their response in terms of hot electron excitation capabilities. Figure 4 presents a summary of these results, showing the local excitation rates for electrons with energies above 1 eV in three representative systems along with the spectra of this magnitude integrated over the surface of the AgPd HNPs. We introduce this threshold to the excess energy of the hot electrons to center our analysis on the excitation of carriers with sufficient kinetic energy to traverse from the metal to the adsorbed reactants. The threshold magnitude was chosen as an approximation for the difference between the Fermi level of the metal and the lowest-unoccupied molecular orbital (LUMO) of the molecule,^43^ with the additional simplifying assumption that the threshold will be the same for both Ag and AgPd alloy so that we can analyze the impact of plasmonic effects separately.

**Figure 4:**
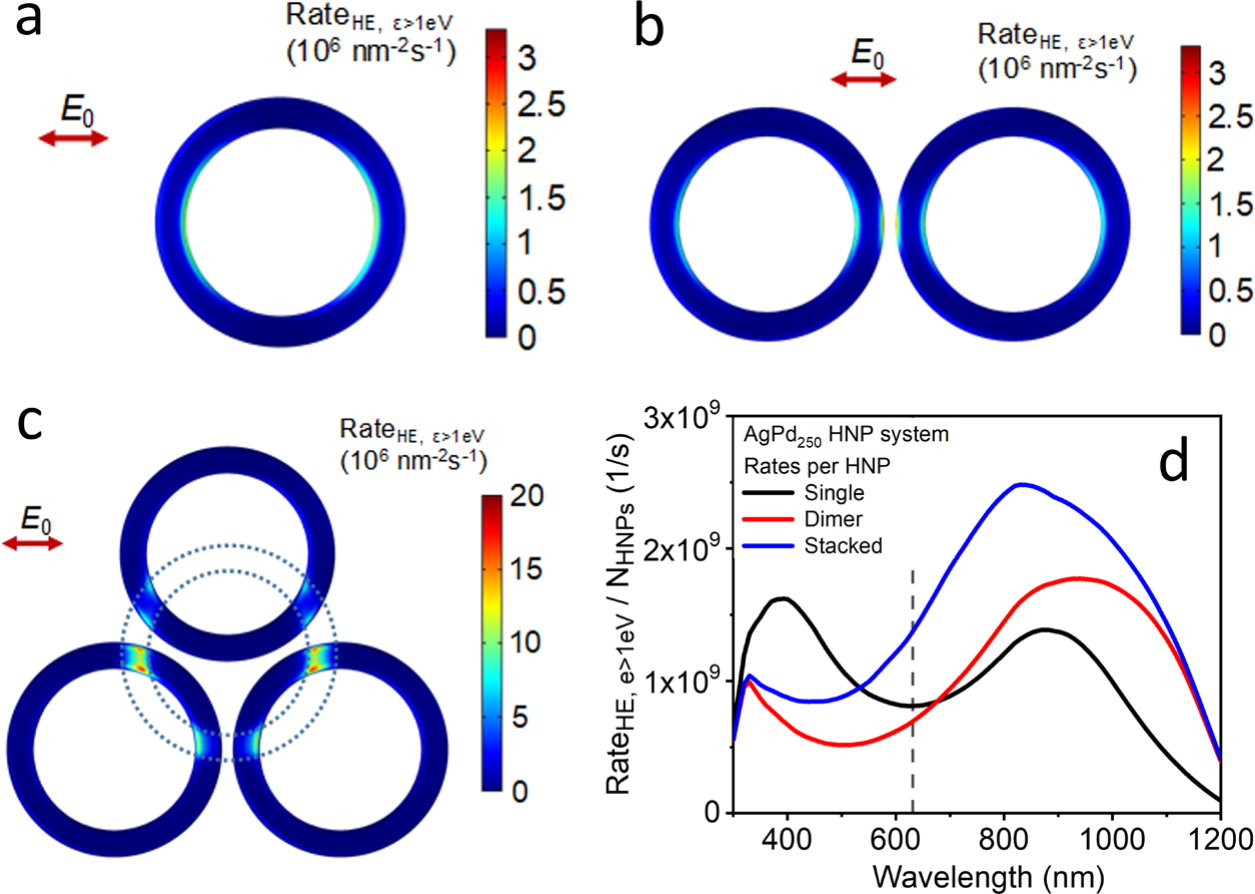
Computational results of different alloyed AgPd HNPs. (a–c) The surface maps show the rates of excitation of hot carriers with energy larger than 1 eV over the Fermi energy, under orthogonally impinging 630 nm illumination, for three isolated systems: (a) single AgPd HNP, (b) dimer, and (c) stacked system of 4 AgPd HNPs, with the top AgPd HNP hidden to reveal the response at the interparticle hot spots. (d) Rates of high-energy hot carriers integrated over the surface of all HNPs in these systems, under unpolarized light, normalized by the number of AgPd HNPs. The vertical dashed line marks the wavelength of the illumination given the surface maps in panels (a–c).

Examining the response of the single AgPd HNP at the experimental irradiation wavelength in Figure 4a, we see that hot carriers are preferentially excited at the internal edges of the ring. This feature can be understood as arising from the larger curvature of the internal edge, in comparison with the outer edge with a larger radius, leading to a stronger local electric field. Although exciting the AgPd HNP at this wavelength does not fully exploit the plasmonic peaks supported by the thickness of the ring, at ∼400 nm, or the whole structure, at ∼840 nm, it provides a rate of hot carrier excitation comparable to those at these peaks. Now, Figure 4b shows another map of the local rate of excitation, in this case for a AgPd HNP dimer, with both AgPd HNPs in the same plane and separated by 2 nm. Examining it reveals that at 630 nm, such simple interparticle coupling does not change much the overall symmetry and peak magnitude of the local high-energy hot carrier excitation rate, although the gap hot spot has a strong contribution. However, in Figure 4d, our computational model indicates that when considering unpolarized light at the same wavelength, the total excitation rates of the dimer do not rise over that of a single HNP, considered on a per-HNP basis. In addition, the excitation rates of AgPd HNP dimers are not influenced by the interparticle distance at 630 nm (Figure S14). This will change when we consider the interaction with additional neighbors.

As mentioned above, Figure 2a shows that the interaction between AgPd HNPs will include more than two resonators in close proximity. We extend our simulations beyond the dimer to bring our models closer to the complexity of the experimental system. We explore the response of the system with two additional AgPd HNPs, first adding a third that breaks the neat dipolar configuration of the dimer, and later adding a fourth AgPd HNP to evaluate the impact of considering the layering of resonators. Such accrual of multiple layers is a very likely situation in our systems due to the repeated deposition process used to load the paper support, and multiple instances of this overlap can be clearly seen in Figure 2a. It is also worth noting that, contrary to what we see for the flat Ag NPs, the AgPd HNPs do not tend to stack neatly (Figure S4); thus, we chose a multilayer AgPd HNP arrangement with resonators offset in the vertical direction. Moreover, we expect this offset to be a significant factor in terms of the overall generation of hot electrons due to the interparticle interaction. We analyze this aspect in Figure S15 in a simplified system with two AgPd HNPs, showing that increasing the offset between two stacked AgPd HNPs strongly increases the rate of excitation of hot carriers, even when their extinction shows relatively small changes. Overall, what we see in Figure 4d is that the combined effect of the in-plane neighbor interaction and vertical stacking makes the aggregated and layered ensemble a more promising photocatalytic system than the single AgPd HNPs. This is so at the wavelength chosen for the experiment, but more so at longer wavelengths, suggesting its promise as a photocatalyst under broadband light. However, since we aim to exploit the UV and high-energy visible ranges, our theoretical results identify the single AgPd HNP as being better suited for driving photocatalysis.

In summary, we have designed a robust and efficient plasmon-enhanced photocatalyst platform, that is, AgPd HNP-loaded cellulose filter papers, toward the C–C homocoupling reaction. The synergistic effect of alloyed AgPd nanoring structures shows excellent LSPR properties for efficient hot electron generation and selectivity for the Ullman C–C coupling reaction. Additionally, these substrates allowed us to perform a time-resolved SERS analysis of the reaction, unraveling the effects of laser power, laser line, and temperature. Our findings demonstrated that hot electrons are the key impact on the plasmon-enhanced C–C coupling reaction process rather than the Joule effect. In addition, the results suggest that the incorporation of Pd into an alloy with plasmonic properties may enhance its catalytic performance under light irradiation due to the collection of LSPR-induced hot electrons. This work provides a strategy for the rational design of in situ SERS substrates and proposes a mechanism for the C–C homocoupling reactions.

## Supporting Information Available

Experimental materials and characterization methods; XRD pattern of Ag NPs (a) and AgPd HNPs (b); time-resolved SERS spectra of the C–C cross-coupling reaction on AgPd HNPs doped paper substrate under different 532 nm laser power densities; computational sampling of the effect of horizontal offset in stacked HNPs; Raman assignment of the different molecules; and COMSOL calculations (PDF)

## References

[ref1] Liz-Marzan L. M.; Murphy C. J.; Wang J. F. Nanoplasmonics. Chem. Soc. Rev. 2014, 43, 3820–3822. 10.1039/c4cs90026j. 24728184

[ref2] Scholl J. A.; Koh A. L.; Dionne J. A. Quantum plasmon resonances of individual metallic nanoparticles. Nature 2012, 483, 421–427. 10.1038/nature10904. 22437611

[ref3] Zheng G. C.; Mourdikoudis S.; Zhang Z. C. Plasmonic Metallic Heteromeric Nanostructures. Small 2020, 16, 2002588 10.1002/smll.202002588. 32762017

[ref4] Cortes E.; Besteiro L. V.; Alabastri A.; Baldi A.; Tagliabue G.; Demetriadou A.; Narang P. Challenges in Plasmonic Catalysis. ACS Nano 2020, 14, 16202–16219. 10.1021/acsnano.0c08773. 33314905

[ref5] Linic S.; Aslam U.; Boerigter C.; Morabito M. Photochemical transformations on plasmonic metal nanoparticles. Nat. Mater. 2015, 14, 567–576. 10.1038/nmat4281. 25990912

[ref6] Linic S.; Christopher P.; Ingram D. B. Plasmonic-metal nanostructures for efficient conversion of solar to chemical energy. Nat. Mater. 2011, 10, 911–921. 10.1038/nmat3151. 22109608

[ref7] Kale M. J.; Avanesian T.; Christopher P. Direct Photocatalysis by Plasmonic Nanostructures. ACS Catal. 2014, 4, 116–128. 10.1021/cs400993w.

[ref8] Ng C.; Cadusch J. J.; Dligatch S.; Roberts A.; Davis T. J.; Mulvaney P.; Gomez D. E. Hot Carrier Extraction with Plasmonic Broadband Absorbers. ACS Nano 2016, 10, 4704–4711. 10.1021/acsnano.6b01108. 26982625

[ref9] Guo J.; Zhang Y.; Shi L.; Zhu Y. F.; Mideksa M. F.; Hou K.; Zhao W. S.; Wang D. W.; Zhao M. T.; Zhang X. F.; Lv J. W.; Zhang J. Q.; Wang X. L.; Tang Z. Y. Boosting Hot Electrons in Hetero-superstructures for Plasmon-Enhanced Catalysis. J. Am. Chem. Soc. 2017, 139, 17964–17972. 10.1021/jacs.7b08903. 29155572

[ref10] Zhang H.; Wei J.; Zhang X. G.; Zhang Y. J.; Radjenovica P. M.; Wu D. Y.; Pan F.; Tian Z. Q.; Li J. F. Plasmon-Induced Interfacial Hot-Electron Transfer Directly Probed by Raman Spectroscopy. Chem 2020, 6, 689–702. 10.1016/j.chempr.2019.12.015.

[ref11] Wu K.; Chen J.; McBride J. R.; Lian T. Efficient hot-electron transfer by a plasmon-induced interfacial charge-transfer transition. Science 2015, 349, 632–635. 10.1126/science.aac5443. 26250682

[ref12] Yang T. H.; Ahn J.; Shi S.; Wang P.; Gao R. Q.; Qin D. Noble-Metal Nanoframes and Their Catalytic Applications. Chem. Rev. 2021, 121, 796–833. 10.1021/acs.chemrev.0c00940. 33275408

[ref13] Li J. M.; Liu J. Y.; Yang Y.; Qin D. Bifunctional Ag@Pd-Ag Nanocubes for Highly Sensitive Monitoring of Catalytic Reactions by Surface-Enhanced Raman Spectroscopy. J. Am. Chem. Soc. 2015, 137, 7039–7042. 10.1021/jacs.5b03528. 25996238

[ref14] Zhang X.; Li X. Q.; Reish M. E.; Zhang D.; Su N. Q.; Gutierrez Y.; Moreno F.; Yang W. T.; Everitt H. O.; Liu J. Plasmon-Enhanced Catalysis: Distinguishing Thermal and Nonthermal Effects. Nano Lett. 2018, 18, 1714–1723. 10.1021/acs.nanolett.7b04776. 29438619

[ref15] Baffou G.; Bordacchini I.; Baldi A.; Quidant R. Simple experimental procedures to distinguish photothermal from hot-carrier processes in plasmonics. Light: Sci. Appl. 2020, 9, 108 10.1038/s41377-020-00345-0. 32612818 PMC7321931

[ref16] Olsen T.; Schiotz J. Origin of Power Laws for Reactions at Metal Surfaces Mediated by Hot Electrons. Phys. Rev. Lett. 2009, 103, 238301 10.1103/PhysRevLett.103.238301. 20366180

[ref17] Zheng Z. K.; Tachikawa T.; Majima T. Plasmon-Enhanced Formic Acid Dehydrogenation Using Anisotropic Pd–Au Nanorods Studied at the Single-Particle Level. J. Am. Chem. Soc. 2015, 137, 948–957. 10.1021/ja511719g. 25543832

[ref18] Jain P. K. Taking the Heat Off of Plasmonic Chemistry. J. Phys. Chem. C 2019, 123, 24347–24351. 10.1021/acs.jpcc.9b08143.

[ref19] Cortes E.; Grzeschik R.; Maier S. A.; Schlucker S. Experimental characterization techniques for plasmon-assisted chemistry. Nat. Rev. Chem. 2022, 6, 259–274. 10.1038/s41570-022-00368-8. 37117871

[ref20] Qiu L.; Pang G. A.; Zheng G. C.; Bauer D.; Wieland K.; Haisch C. Kinetic and Mechanistic Investigation of the Photocatalyzed Surface Reduction of 4-Nitrothiophenol Observed on a Silver Plasmonic Film via Surface-Enhanced Raman Scattering. ACS Appl. Mater. Interfaces 2020, 12, 21133–21142. 10.1021/acsami.0c05977. 32286058

[ref21] Zhan C.; Wang Z. Y.; Zhang X. G.; Chen X. J.; Huang Y. F.; Hu S.; Li J. F.; Wu D. Y.; Moskovits M.; Tian Z. Q. Interfacial Construction of Plasmonic Nanostructures for the Utilization of the Plasmon-Excited Electrons and Holes. J. Am. Chem. Soc. 2019, 141, 8053–8057. 10.1021/jacs.9b02518. 31070906

[ref22] Boerigter C.; Campana R.; Morabito M.; Linic S. Evidence and implications of direct charge excitation as the dominant mechanism in plasmon-mediated photocatalysis. Nat. Commun. 2016, 7, 10545 10.1038/ncomms10545. 26817619 PMC4738363

[ref23] Langer J.; de Aberasturi D. J.; Aizpurua J.; Alvarez-Puebla R. A.; Auguie B.; Baumberg J. J.; Bazan G. C.; Bell S. E. J.; Boisen A.; Brolo A. G.; Choo J.; Cialla-May D.; Deckert V.; Fabris L.; Faulds K.; de Abajo F. J. G.; Goodacre R.; Graham D.; Haes A. J.; Haynes C. L.; Huck C.; Itoh T.; Ka M.; Kneipp J.; Kotov N. A.; Kuang H.; Le Ru E. C.; Lee H. K.; Li J. F.; Ling X. Y.; Maier S. A.; Mayerhofer T.; Moskovits M.; Murakoshi K.; Nam J. M.; Nie S.; Ozaki Y.; Pastoriza-Santos I.; Perez-Juste J.; Popp J.; Pucci A.; Reich S.; Ren B.; Schatz G. C.; Shegai T.; Schlucker S.; Tay L. L.; Thomas K. G.; Tian Z. Q.; Van Duyne R. P.; Vo-Dinh T.; Wang Y.; Willets K. A.; Xu C.; Xu H.; Xu Y.; Yamamoto Y. S.; Zhao B.; Liz-Marzan L. M. Present and Future of Surface-Enhanced Raman Scattering. ACS Nano 2020, 14, 28–117. 10.1021/acsnano.9b04224. 31478375 PMC6990571

[ref24] Guselnikova O.; Lim H.; Kim H. J.; Kim S. H.; Gorbunova A.; Eguchi M.; Postnikov P.; Nakanishi T.; Asahi T.; Na J.; Yamauchi Y. New Trends in Nanoarchitectured SERS Substrates: Nanospaces, 2D Materials, and Organic Heterostructures. Small 2022, 18, 2107182 10.1002/smll.202107182. 35570326

[ref25] Lim H.; Kim D.; Kim Y.; Nagaura T.; You J.; Kim J.; Kim H. J.; Na J.; Henzie J.; Yamauchi Y. A mesopore-stimulated electromagnetic near-field: electrochemical synthesis of mesoporous copper films by micelle self-assembly. J. Mater. Chem. A 2020, 8, 21016–21025. 10.1039/D0TA06228F.

[ref26] Shi S.; Qin D. Bifunctional Metal Nanocrystals for Catalyzing and Reporting on Chemical Reactions. Angew. Chem., Int. Ed. 2020, 59, 3782–3792. 10.1002/anie.201909615. 31529749

[ref27] Li Y. L.; Hu Y. F.; Shi F. X.; Li H. X.; Xie W.; Chen J. C–H Arylation on Nickel Nanoparticles Monitored by In Situ Surface-Enhanced Raman Spectroscopy. Angew. Chem., Int. Ed. 2019, 58, 9049–9053. 10.1002/anie.201902825. 31025515

[ref28] Xu L.; Luo Z. M.; Fan Z. X.; Yu S. J.; Chen J. Z.; Liao Y. S.; Xue C. Controllable Galvanic Synthesis of Triangular Ag–Pd Alloy Nanoframes for Efficient Electrocatalytic Methanol Oxidatio. Chem. - Eur. J. 2015, 21, 8691–8695. 10.1002/chem.201406677. 25925988

[ref29] Xiong Y. J.; Xia Y. N. Shape-Controlled Synthesis of Metal Nanostructures: The Case of Palladium. Adv. Mater. 2007, 19, 3385–3391. 10.1002/adma.200701301.

[ref30] Garcia-Lojo D.; Nunez-Sanchez S.; Gomez-Grana S.; Grzelczak M.; Pastoriza-Santos I.; Perez-Juste J.; Liz-Marzan L. M. Plasmonic Supercrystals. Acc. Chem. Res. 2019, 52, 1855–1864. 10.1021/acs.accounts.9b00213. 31243968

[ref31] Solis D. M.; Taboada J. M.; Obelleiro F.; Liz-Marzan L. M.; de Abajo F. J. G. Optimization of Nanoparticle-Based SERS Substrates through Large-Scale Realistic Simulations. ACS Photonics 2017, 4, 329–337. 10.1021/acsphotonics.6b00786. 28239616 PMC5319398

[ref32] Zheng G. C.; Kaefer K.; Mourdikoudis S.; Polavarapu L.; Vaz B.; Cartmell S. E.; Bouleghlimat A.; Buurma N. J.; Yate L.; de Lera A. R.; Liz-Marzan L. M.; Pastoriza-Santos I.; Perez-Juste J. Palladium Nanoparticle-Loaded Cellulose Paper: A Highly Efficient, Robust, and Recyclable Self-Assembled Composite Catalytic System. J. Phys. Chem. Lett. 2015, 6, 230–238. 10.1021/jz5024948. 26263455

[ref33] Zheng G. C.; Polavarapu L.; Liz-Marzan L. M.; Pastoriza-Santos I.; Perez-Juste J. Gold nanoparticle-loaded filter paper: a recyclable dip-catalyst for real-time reaction monitoring by surface enhanced Raman scattering. Chem. Commun. 2015, 51, 4572–4575. 10.1039/C4CC09466B. 25578310

[ref34] Sjöström E. The origin of charge on cellulosic fibers. Nord. Pulp Pap. Res. J. 1989, 4, 90–93. 10.3183/npprj-1989-04-02-p090-093.

[ref35] Zhao H.; Li C. F.; Yong X.; Kumar P.; Palma B.; Hu Z. Y.; Van Tendeloo G.; Siahrostami S.; Larter S.; Zheng D.; Wang S.; Chen Z.; Kibria M. G.; Hu J. Coproduction of hydrogen and lactic acid from glucose photocatalysis on band-engineered Zn1-xCdxS homojunction. iScience 2021, 24, 102109 10.1016/j.isci.2021.102109. 33615204 PMC7881236

[ref36] Amatore C.; Le Duc G.; Jutand A. Mechanism of Palladium-Catalyzed Suzuki–Miyaura Reactions: Multiple and Antagonistic Roles of Anionic “Bases” and Their Countercations. Chem. - Eur. J. 2013, 19, 10082–10093. 10.1002/chem.201300177. 23787914

[ref37] Yang H.; Chu D. Z.; Jiao L. Aromatization modulates the activity of small organic molecules as promoters for carbon–halogen bond activation. Chem. Sci. 2018, 9, 1534–1539. 10.1039/C7SC04450J. 29675197 PMC5887235

[ref38] Taladriz-Blanco P.; Herves P.; Perez-Juste J. Supported Pd Nanoparticles for Carbon–Carbon Coupling Reactions. Top. Catal. 2013, 56, 1154–1170. 10.1007/s11244-013-0082-6.

[ref39] Dinh C. T.; Yen H.; Kleitz F.; Do T. O. Three-Dimensional Ordered Assembly of Thin-Shell Au/TiO2 Hollow Nanospheres for Enhanced Visible-Light-Driven Photocatalysis. Angew. Chem., Int. Ed. 2014, 53, 6618–6623. 10.1002/anie.201400966. 24737715

[ref40] Hartland G. V.; Besteiro L. V.; Johns P.; Govorov A. O. What’s so Hot about Electrons in Metal Nanoparticles?. ACS Energy Lett. 2017, 2, 1641–1653. 10.1021/acsenergylett.7b00333.

[ref41] Besteiro L. V.; Kong X. T.; Wang Z. M.; Hartland G.; Govorov A. O. Understanding Hot-Electron Generation and Plasmon Relaxation in Metal Nanocrystals: Quantum and Classical Mechanisms. ACS Photonics 2017, 4, 2759–2781. 10.1021/acsphotonics.7b00751.

[ref42] Santiago E. Y.; Besteiro L. V.; Kong X. T.; Correa-Duarte M. A.; Wang Z. M.; Govorov A. O. Efficiency of Hot-Electron Generation in Plasmonic Nanocrystals with Complex Shapes: Surface-Induced Scattering, Hot Spots, and Interband Transitions. ACS Photonics 2020, 7, 2807–2824. 10.1021/acsphotonics.0c01065.

[ref43] Nan L.; Giráldez-Martínez J.; Stefancu A.; Zhu L.; Liu M.; Govorov A. O.; Besteiro L. V. Investigating Plasmonic Catalysis Kinetics on Hot-Spot Engineered Nanoantennae. Nano Lett. 2023, 23, 2883–2889. 10.1021/acs.nanolett.3c00219. 37001024

