# Supplementary material for: Differentiating Plasmon-Enhanced Chemical Reactions on AgPd Hollow Nanoplates through Surface-Enhanced Raman Spectroscopy
Source: ACS Catal. 2024 Apr 17;14(9):6799–806. doi: 10.1021/acscatal.3c06253 (PMC11075454; doi:10.1021/acscatal.3c06253)
Supplement: Supplementary file 1 — cs3c06253_si_001.pdf [file cs3c06253_si_001.pdf]

Supporting information

for

Differentiating plasmon-enhanced chemical reactions on AgPd hollow nanoplates through Surface-enhanced Raman Spectroscopy

Sulin Jiao,[a,b#] Kun Dai,[b#] Lucas V. Besteiro,[c,d] Hongen Gao,[a] Xuan Chem,[a] Weichao Wang,[a] Yuan Zhang,[a] Chuntai Liu,[b] Ignacio Pérez-Juste,[e] Jorge Perez-Juste,[c,e*] Isabel Pastoriza-Santos,[c,e] Guangchao Zheng[a,f*]

[a] Key Laboratory of Materials Physics, Ministry of Education, School of Physics, Zhengzhou University, Zhengzhou 450001, P. R. China

[b] School of Materials Science and Engineering, Key Laboratory of Materials Processing and Mold, Zhengzhou University, Zhengzhou, 450001, P. R. China

[c] CINBIO, Universidade de Vigo, Campus Universitario As Lagoas, Marcosende, 36310 Vigo, Spain

[d] Departamento de Física Aplicada, Universidade de Vigo, Campus Universitario As Lagoas, Marcosende, 36310 Vigo, Spain.

[e] Departamento de Química Física, Universidade de Vigo, Campus Universitario As Lagoas, Marcosende, 36310 Vigo, Spain.

[f] Institute of Quantum Materials and Physics, Henan Academy of Sciences, Zhengzhou 450046, China

Corresponding Authors

Guangchao Zheng- S Key Laboratory of Materials Physics, Ministry of Education, School of Physics, Zhengzhou University, Zhengzhou 450001, P. R. China. Email: gczheng@zzu.edu.cn.

Jorge Pérez-Juste-CINBIO, Universidade de Vigo, Departamento de Química Física, Campus Universitario As Lagoas, Marcosende, 36310 Vigo, Spain. Email: juste@uvigo.es.

EXPERIMENTAL SECTION

Materials and reagents. $AgNO_3$, Na_2PdCl_4, $NaBH_4$, hexadecyltrimethylammonium bromide (CTAB), L-ascorbic acid (AA), NaOH, 4-bromothiophenol (4-BTP), 4,4'-biphenyldithiol (4,4'-BPDT) and sodium thiophenoxide (TP) are all from Adamas-beta. All reagents and solvents were used directly as received without any additional purification.

Synthesis of Ag nanoplates. Silver nanoplates (Ag NPs) were synthesized via seed-mediated approaches according to the previous report.[1] First, silver seeds were produced by introducing 300 μL fresh $NaBH_4$ solution (10 mM) into 10 ml of aqueous solution containing $AgNO_3$ (5 mM) and CTAB (0.5 mM). The seed solution was kept in the darkness for one hour. Subsequently, a growth solution was prepared by adding 2 mL of $AgNO_3$ (10 mM) and 2 mL of ascorbic acid (0.1 M) into 40 ml of CTAB (10 mM) in water. Finally, 400 μL of as prepared seeds and 400 μL of NaOH (0.1 M) were injected into the growth solution. After finishing the reaction, Ag NPs were centrifuged (rpm: 8000; 15 min) and redispersed in 45 mL of water.

Synthesis of alloyed AgPd hollow nanoplates. 5 ml of ascorbic acid (0.1 M) were added to the Ag NP solution with vigorous stirring. Then different amounts (50 or 250 μL) of $NaPdCl_4$ (10 mM) were added to the solution to control the morphology of the ring. After stirring for three hours, AgPd hollow nanoplates (HNPs) were centrifuged (8000 rpm; 15 min) and the precipitate was redispersed in 5 mL of water.

Preparation of Ag nanoplates and AgPd hollow nanoplates-loaded filter paper. Cellulose filter papers were cut into squares (1 cm × 1 cm). Subsequently, each paper square was briefly dipped into the as-prepared colloids of Ag NPs or AgPd HNPs, and then allowed to dry at 60 °C. The dipping process was repeated up to 6 times. Finally, the loaded filter paper was dried in an oven.

Characterizations.
The ultraviolet-visible-NIR (UV-vis-NIR) absorption spectrum was carried out by the UV-1900 (SHIMADZU). The reflectance spectrum was performed by the UV-3600 with integrating sphere (SHIMADZU). The nanostructures were characterized by scanning electron microscopy (SEM, JEOL-6700F), transmission electron microscopy (TEM, JEM-2010, 200 kV), and high-resolution transmission electron microscopy (HRTEM, JEM-2100F, 200 kV). The composition and phase of the nanostructures were analyzed by an x-ray diffractometer (Bruker D8 ADVANCE), which operated at a voltage of 40 kV. Raman spectra were recorded by using confocal Raman spectroscopy (HORIBA LabRAM HR Evolution) at ambient temperature.

In situ SERS monitoring of catalytic experiments. The in-situ SERS substrates were immersed into 2 mL probe molecules (1 mM of 4-BTP, TP or 4,4'-BPDT) overnight.

Next, added 100 μL sodium hydroxide (NaOH, 1 M) dropwise on the substrates and then tested with Horiba Raman spectroscopy. SERS spectra were collected directly from the substrate at different reaction conditions (acquisition time 15 s (one minute for each line), objective 50x LWD, grating 1800 gr/mm). All reactions are performed in a humid environment. To ensure that the NP-loaded cellulose substrate is placed on a petri dish with a thin water layer. For temperature-dependent SERS measurements, substrates were put into a closed chamber to maintain a constant temperature and SERS detection.

SERS peak analysis: Under the same experimental conditions, the peak areas obtained from the three commercial components (4-BTP, TP, and 4,4'-BPDT) were compared to correct and calibrate the peak areas in SERS monitoring for calculating the relative concentration of the three species during the catalytic reaction. Quantitative information on the relative concentration of 4-BTP, TP, and 4,4'-BPDT was extracted by comparing the peak areas of their characteristic bands at 1560, 1573, and 1585 cm^{-1}, respectively.

Computational methods. Geometrical optimization of simple models of TP, 4-BTP and 4,4'-BPDT homolitically bonded to one or two Ag atoms were performed employing density functional theory (DFT) at the M062X level with the 6-311++G** basis set for C, H and S atoms and LANL2DZ and its corresponding pseudopotential for Ag atoms. The obtained structures were characterized as energy minima by computing their vibrational frequencies. All calculations were performed using Gaussian16.[2] Assignment of the theoretical Raman spectrum to molecular vibrational modes, scaled by 0.9567, was performed by visual inspection of each vibrational mode combined with the results from VEDA, a program that generates an optimized set of internal coordinates based on the molecular structure and fits a potential energy distribution for the quantitative analysis of vibrational spectra.[3]

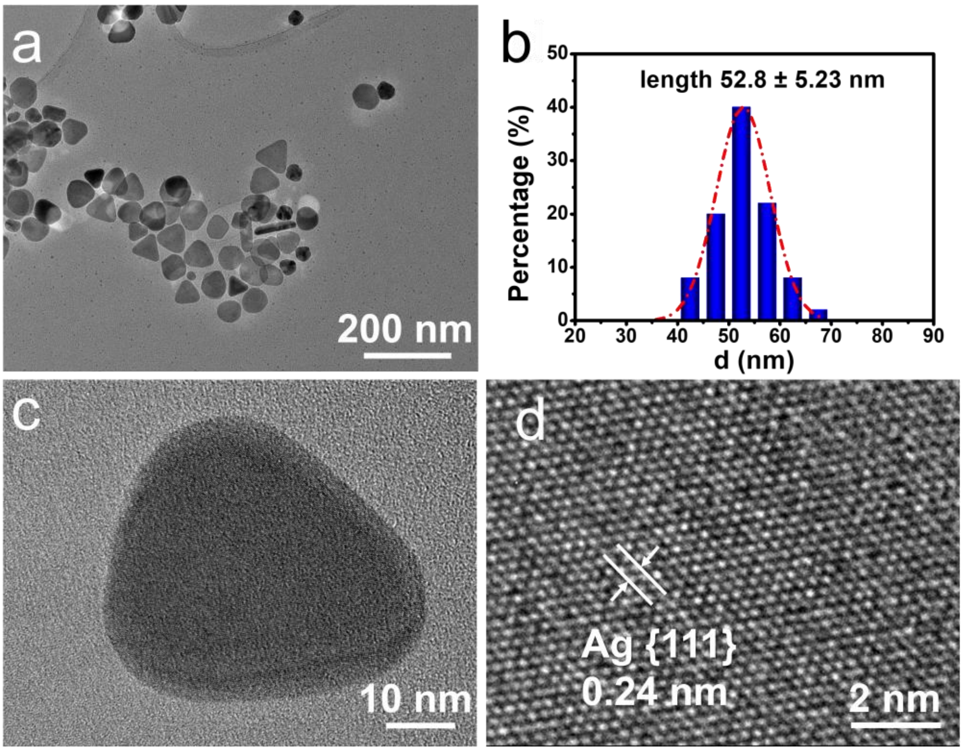

Figure S1. (a) Representative TEM image of Ag NPs. (b) Size distribution histogram. (b) TEM of single Ag NP. (c) High-resolution transmission electron microscopy (HRTEM) image of Ag NP showing the characteristic Ag (111) planes assigned by the lattice fringes with 0.24 nm spacing.

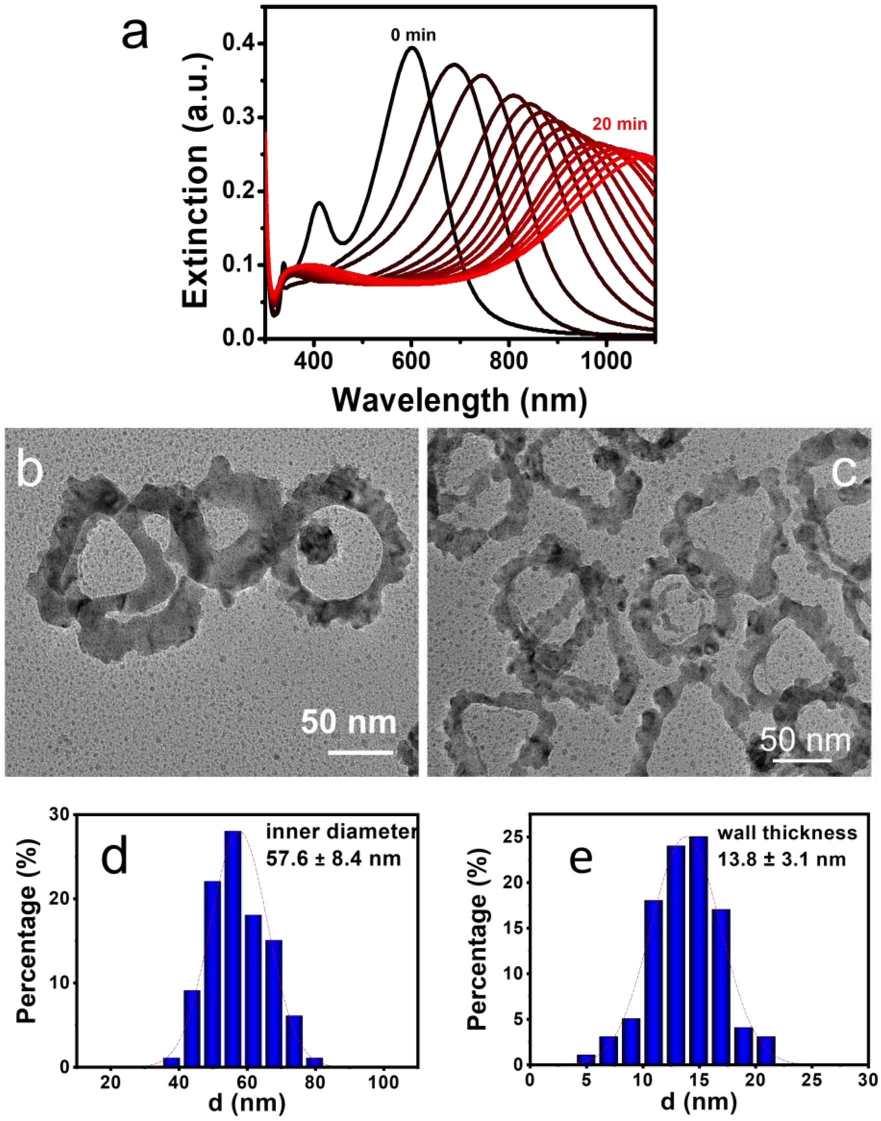

Figure S2. (a) Time evolution UV-vis-NIR extinction spectra of the galvanic replacement of Ag nanoplates upon the addition of the palladium salt precursor. (b-c) Representative TEM images showing the morphology of the particles after the galvanic replacement reaction. (c) Size distribution histogram of the inner diameter of the AgPd HNPs. (d) Size distribution histogram of the wall thickness of the final AgPd HNPs.

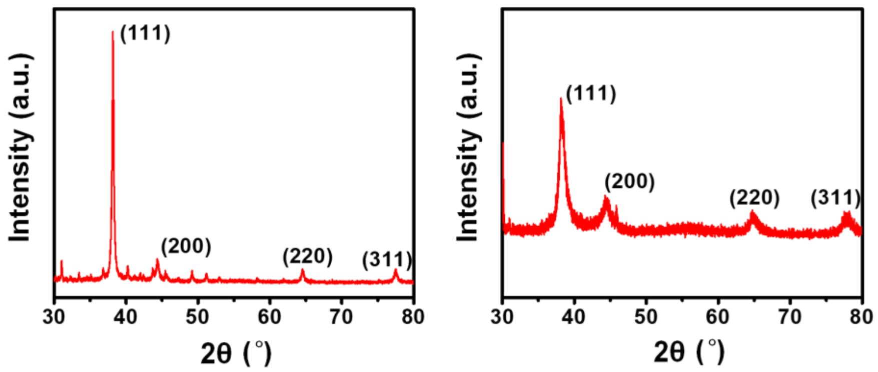

Figure S3. XRD pattern of Ag NPs (a) and AgPd HNPs (b).

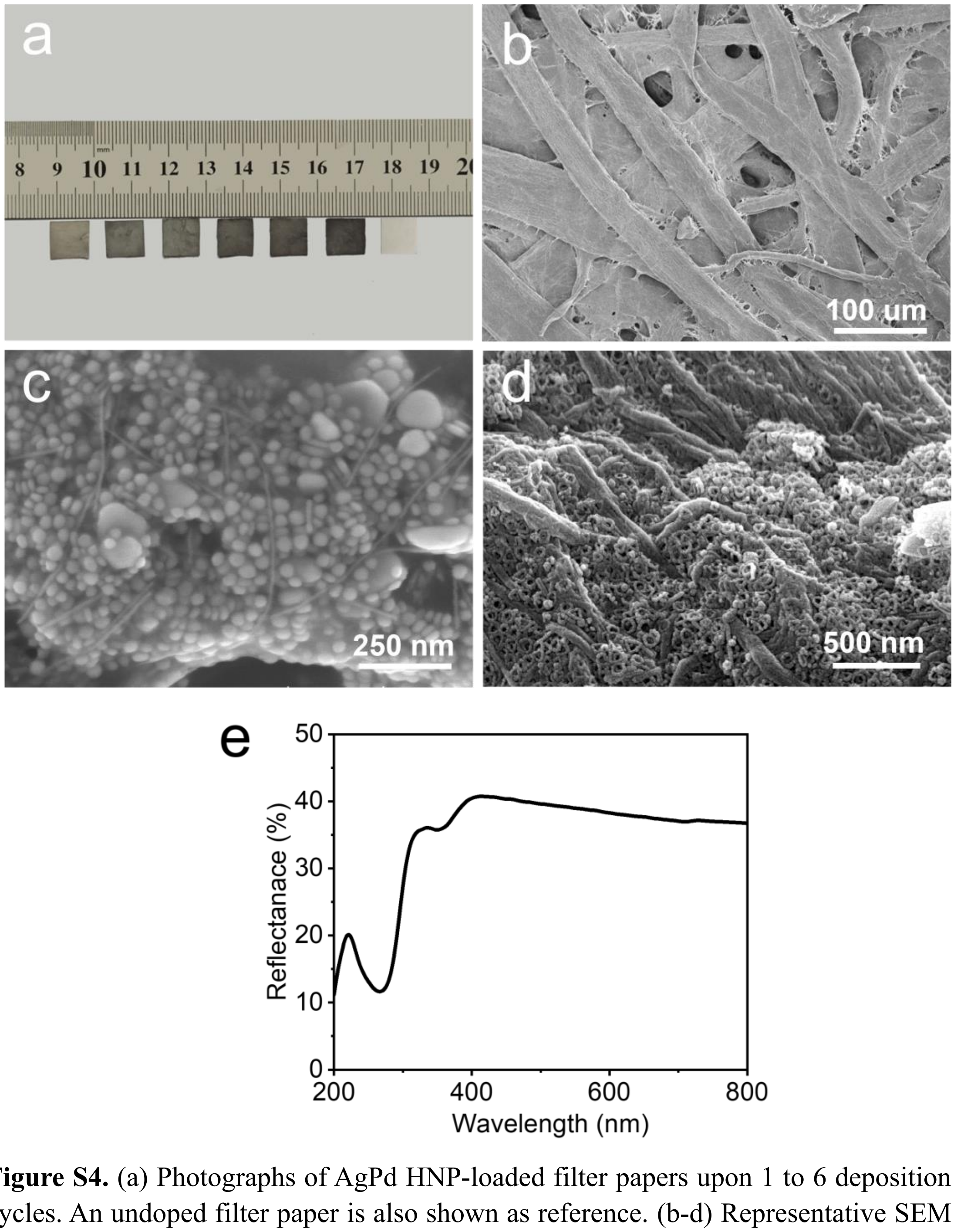

Figure S4. (a) Photographs of AgPd HNP-loaded filter papers upon 1 to 6 deposition cycles. An undoped filter paper is also shown as reference. (b-d) Representative SEM image of an undoped filter paper (b) and Ag NP-loaded filter paper (c) and AgPd HNP-loaded filter paper (d). (e) Optical properties (reflectance) of an AgPd HNP-loaded filter papers after 6 deposition cycles.

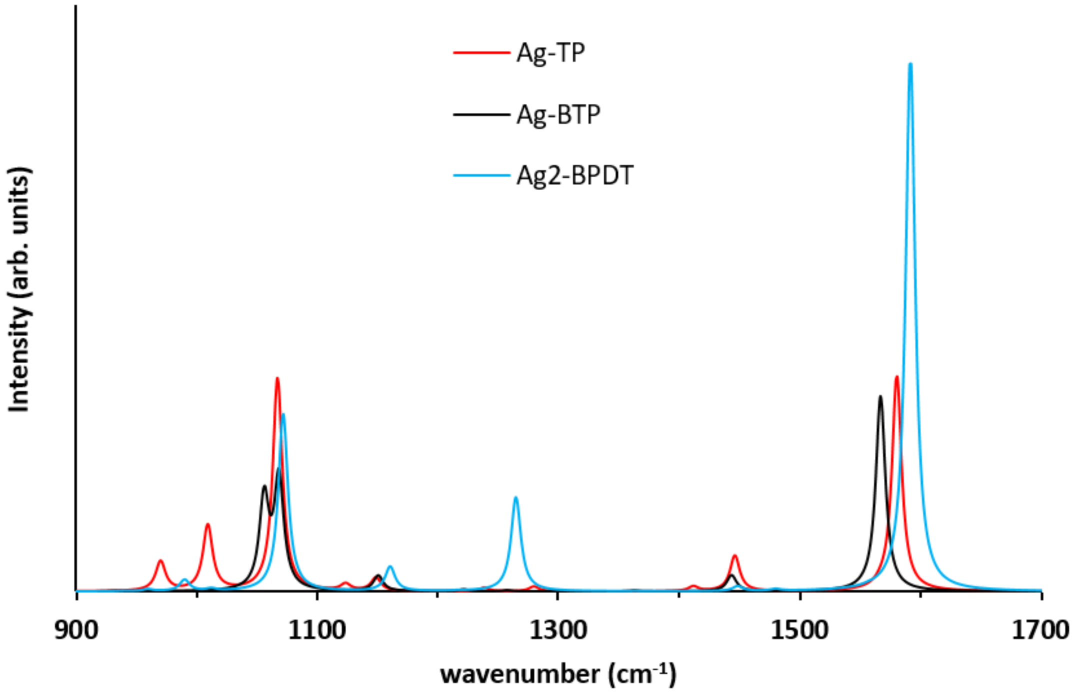

Figure S5. Theoretical Raman spectra of the thiophenol (Ag-TP), bromothiophenol (Ag-4-BTP) and (Ag2-4-BPDT). Vibrational frequencies have been scaled by 0.9675 and Raman intensities scaled to reproduce the experimental intensities.[4] Table S1 shows the theoretical vibrational frequencies and vibrational assignments (including its potential energy distribution).

Raman/SERS spectra are dominated by a signal around 1560 cm^{-1} associated with the symmetric stretching of the aromatic ring and a group of smaller signals around 1000-1100 cm^{-1} originated by mixed modes involving ring deformations and CCH bendings. Theoretical spectra agree very well with the experimental data and the wavenumbers shifts for the signal around 1560 cm^{-1} confirm the evolution of the reaction of BTP to its dimer 4,4'-BDPT or the formation of 4-TP. The signal around 1265 cm^{-1} is only observed for the 4,4'-BDPT species and becomes an additional confirmation of the expected formation of the dimer.

Table S1. Raman assignment of the different molecules

Ag-TP

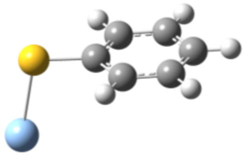

1580.5	C=C symmetric stretching (65%)
1446.3	Ring deformation (28%) + CCH bending (62%)
1148.6	CCH bending (76%)
1067.0	Ring deformation (57%) + CCH bending (23%) [breathing mode]
1009.1	Ring deformation (71%) + CCH bending (12%) [breathing mode]
970.2	Ring deformation (64%) [asymmetric breathing out of plane]

Ag-BTP

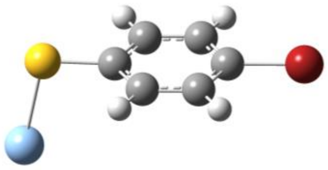

1567.1	C=C symmetric stretching (73%)
1443.6	Ring deformation (32%) + CCH bending (61%)
1150.5	CCH symmetric bending (79%)
1068.2	Ring deformation (46%) + CCH bending (29%) + SC stretching (16%)
1055.9	Ring deformation (82%) [breathing mode]

Ag_2-BPDT

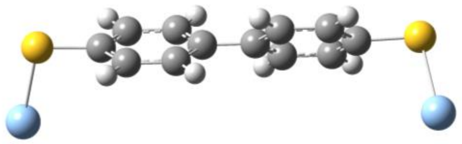

1591.8	C=C symmetric stretching concerted in both rings (65%) + CCH bending (17%)
1448.6	Ring deformation (15%) + CCH bending (56%)
1264.7	Ring deformation (54%) + CCH bending (29%)
1160.4	Ring deformation (15%) + CCH bending (76%)
1071.7	Ring deformation with SC contribution (65%) + CCH bending (16%)

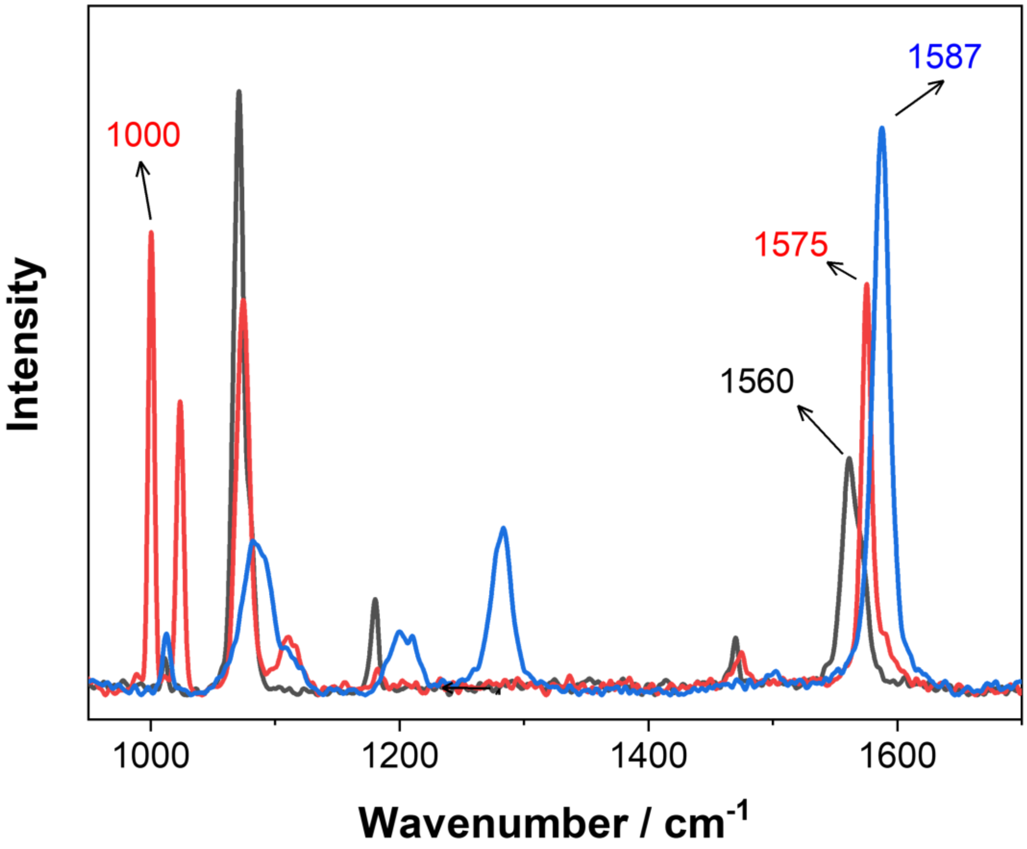

Figure S6. SERS spectra of 4-BTP (black), 4-TP (red), and 4,4'-BPDT (blue) measured on a AgPd HNP-doped substrate. The area ratio of the peaks at 1560, 1575 and 1587 cm^{-1} is 1:1.02:2.70. These ratios were taken into account for calculating the relative concentration of the three components during the catalytic reaction. SERS measurements were carried out with a 633 nm laser line, 50x objective, 0.12 mW/cm^2 laser power density and an acquisition time 15 s.

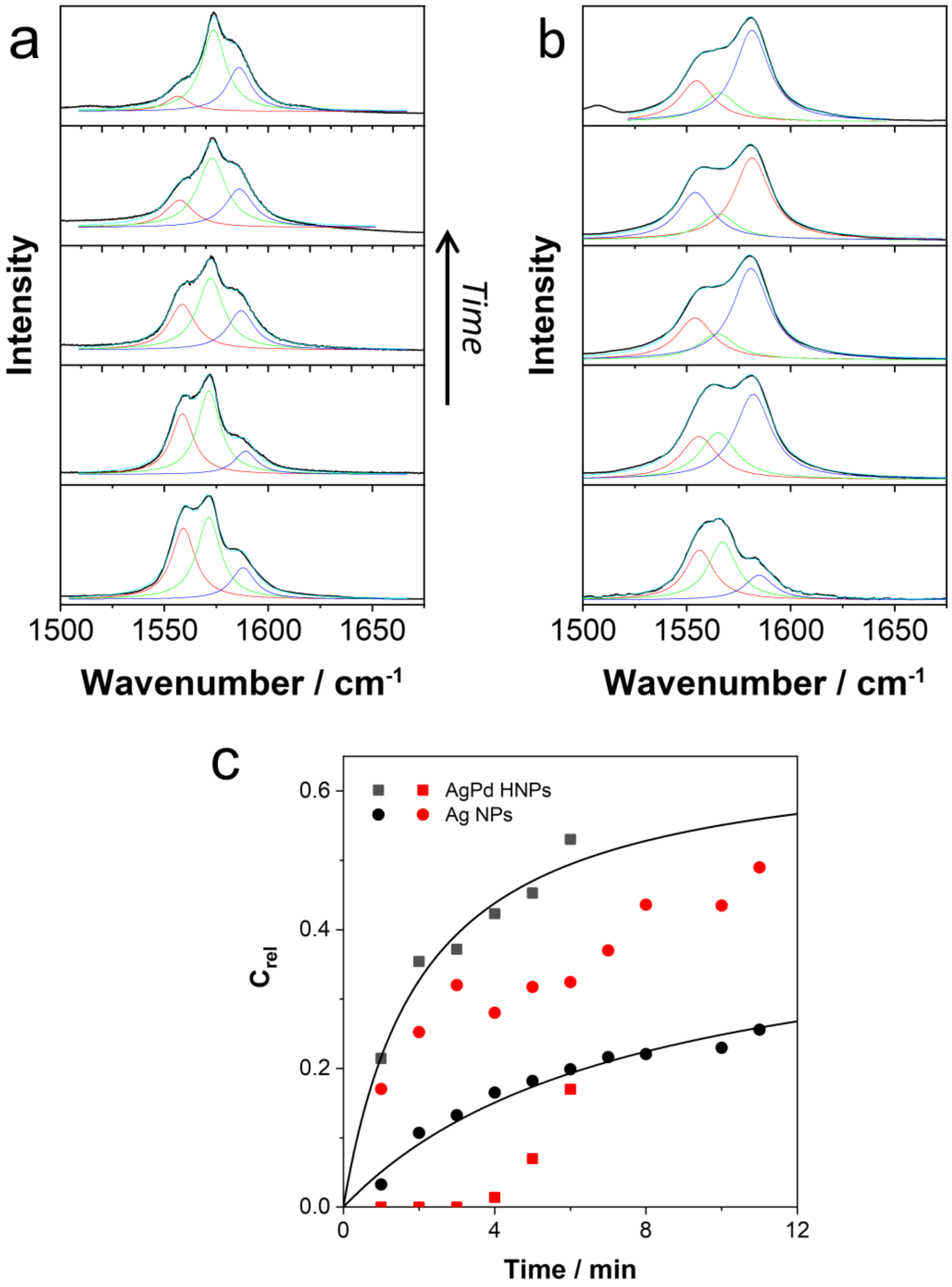

Figure S7. (a-b) Deconvolution of the SERS spectra into the relative contributions of 4-BTP (red), TP (green) and 4,4'-BPDT (blue) during the catalytic reaction on Ag NP (a) and AgPd HNP (b) substrates. (c) Relative concentrations of 4,4'-BPDT (black symbols) and TP (red symbols) on both plasmonic substrates, as indicated. SERS measurements were carried out with a 633 nm laser line, 50x objective, 0.12 mW/cm^2 laser power density and an acquisition time 15 s.

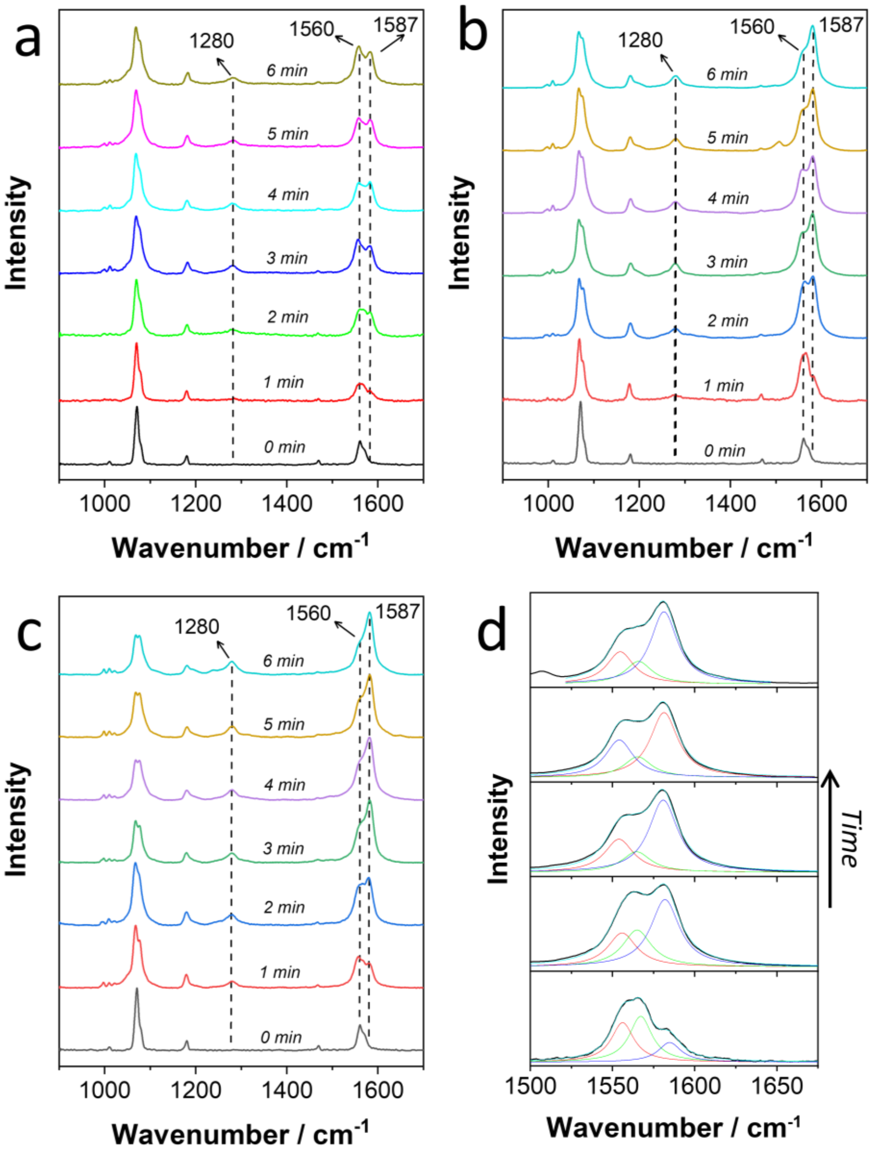

Figure S8. Time-resolved SERS spectra of the C-C cross-coupling reaction on AgPd HNPs doped paper substrate under different 633 nm laser power densities: (a) 0.05 mW/cm^2, (b) 0.12 mW/cm^{2}and (c) 0.38 mW/cm^2. (d) Representative deconvolution of the SERS spectra into the relative contributions of 4-BTP (red), TP (green) and 4,4'-BPDT (blue) during the catalytic reaction shown in (b). SERS measurements were carried out with a 633 nm laser line, 50x objective, and an acquisition time 15 s.

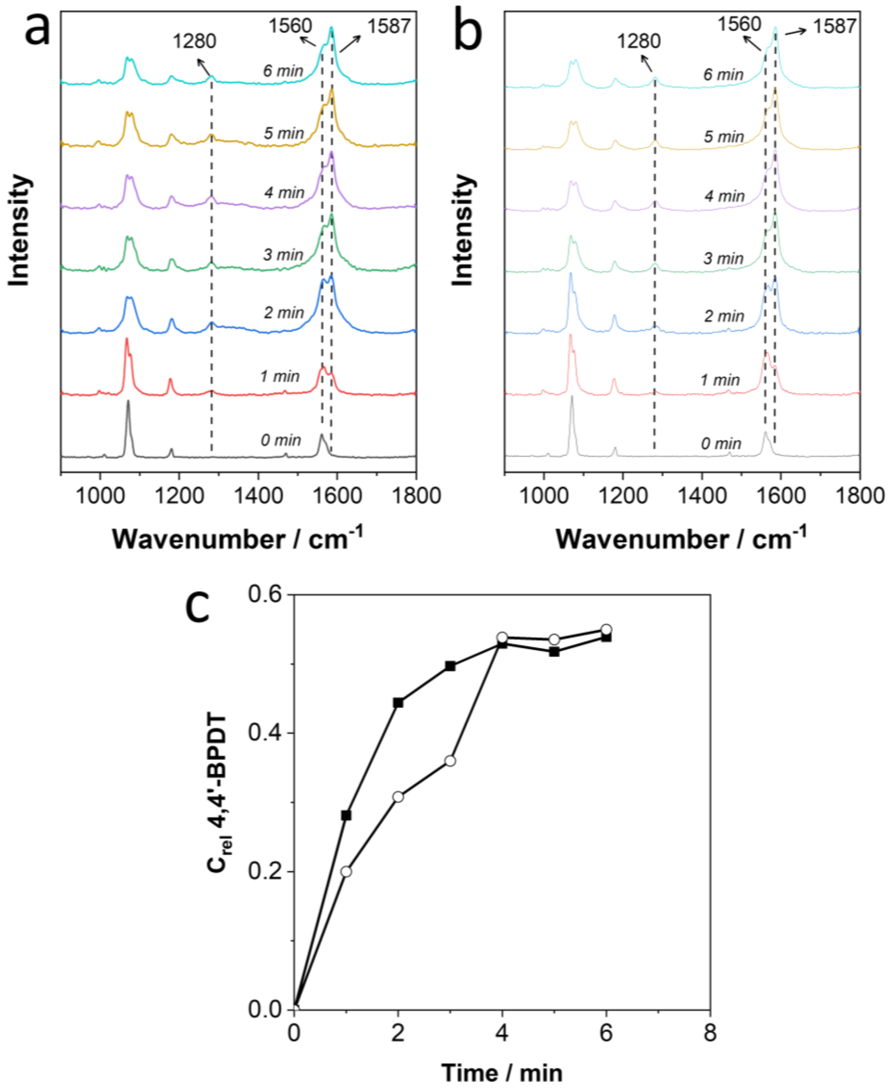

Figure S9. Time-resolved SERS spectra of the C-C cross-coupling reaction on AgPd HNPs doped paper substrate under different 532 nm laser power densities; (a) 0.15 mW/cm^{2}and (b) 0.23 mW/cm^2. (c) Relative concentration of the product 4,4'-BPDT at two 532 nm laser power densities; 0.23 mW/cm^2 (squares) and 0.15 mW/cm^2 (circles). SERS measurements were carried out with a 532 nm laser line, 50x objective and an acquisition time 15 s.

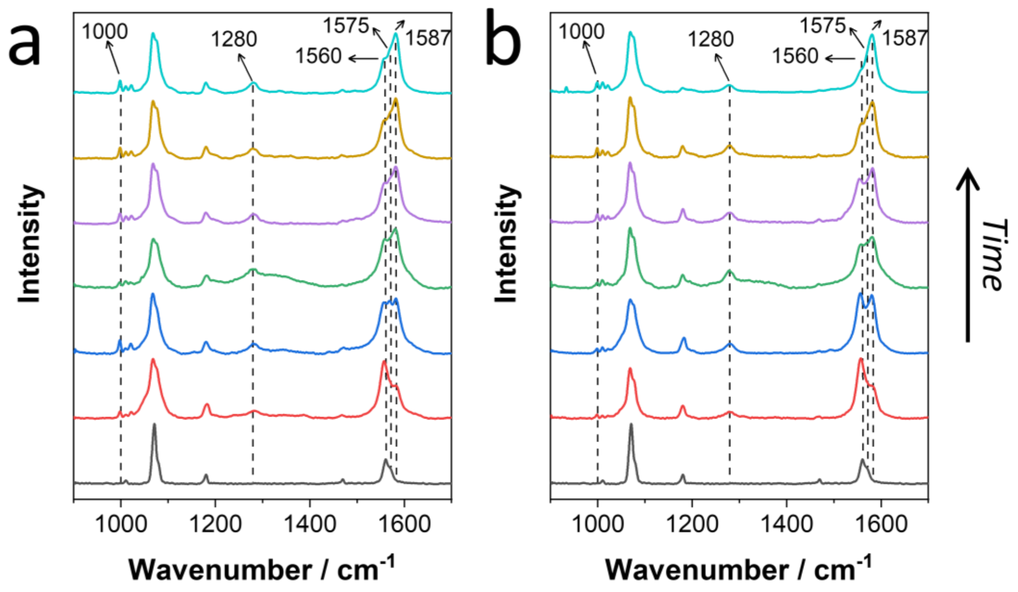

Figure S10. Time-resolved SERS spectra of the reaction on AgPd HNPs doped paper substrate under 633 nm laser power densities (50x objective, 0.12 mW/cm^2 and an acquisition time 15 s) at different temperatures; (a) 313.15 K and (b) 353.15 K.

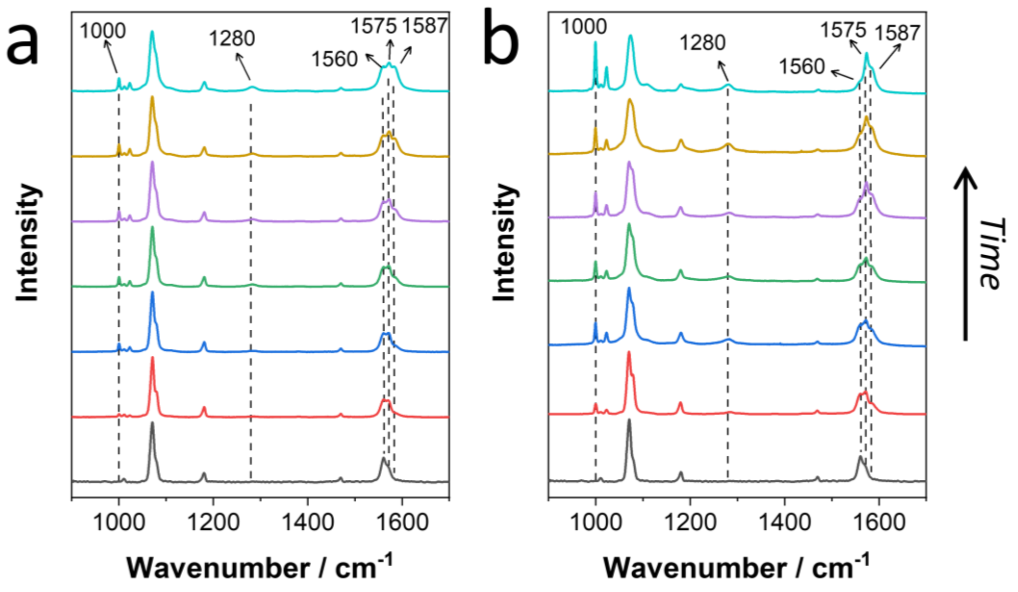

Figure S11. Time-resolved SERS spectra for the catalytic reaction on Ag NP doped substrate in the absence (a) and in the presence of NaOH (pH 14, b), showing a higher 4-TP ratio obtained at basic pH, as demonstrated by the high intensity of the SERS peaks at 1000 and 1575 cm^{-1} characteristic of thiophenol. SERS measurements were carried out with a 633 nm laser line, 50x objective, 0.12 mW/cm^2 laser power density and an acquisition time 15 s.

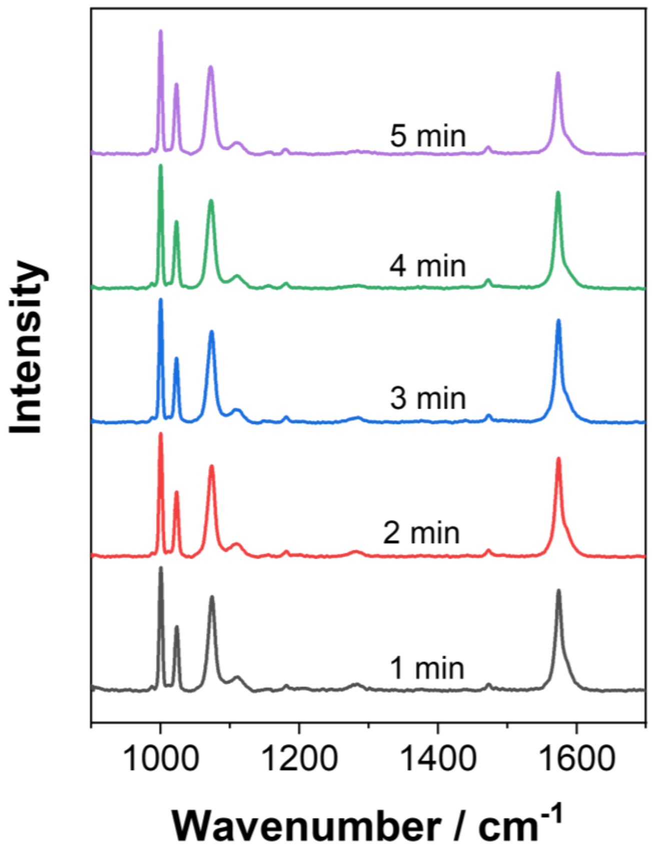

Figure S12. SERS spectra of a 4-TP monolayer measured on a AgPd HNPs doped paper substrate under different 633 nm laser irradiation times. SERS measurements were carried out with a 633 nm laser line, 50x objective, 0.12 mW/cm^2 laser power densityand an acquisition time 15 s.

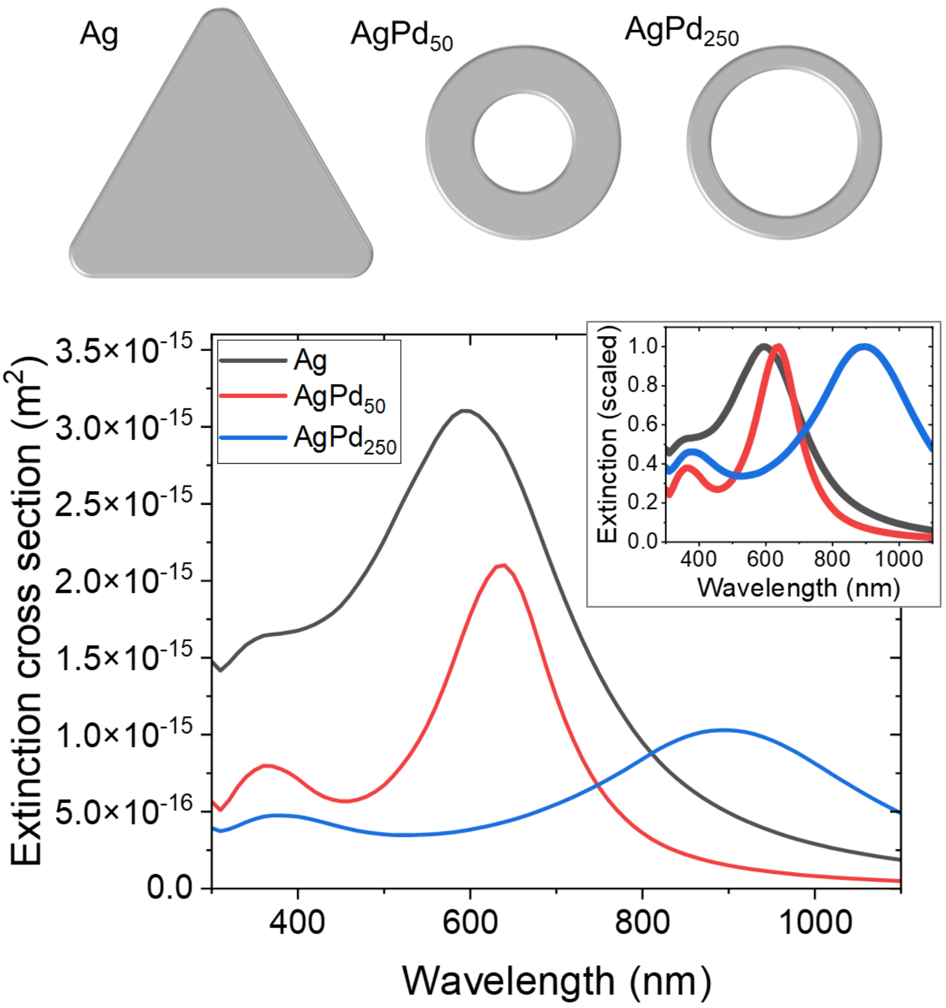

Figure S13. (Top) Theoretical models capturing the progress of the galvanic reduction reaction, going from pure Ag triangular platelets to thin ring alloys. The Ag triangle has a side of 70 nm, with corners rounded with a radius of 5 nm. The alloyed structures have an outer radius of 20 nm and an inner radii of 12 nm and 15 nm, respectively. The three geometries have a thickness of 15 nm and had their edges rounded with a radius of 1 nm to avoid physically unrealistic hot spots at the sharp edges. The permittivity of the materials is taken as pure Ag, 3:1 Ag:Pd mixture and 1:1 Ag:Pd mixture, respectively. (Bottom) Extinction cross section of the simulated models, with the inset showing the same data, scaled to the [0,1] range.

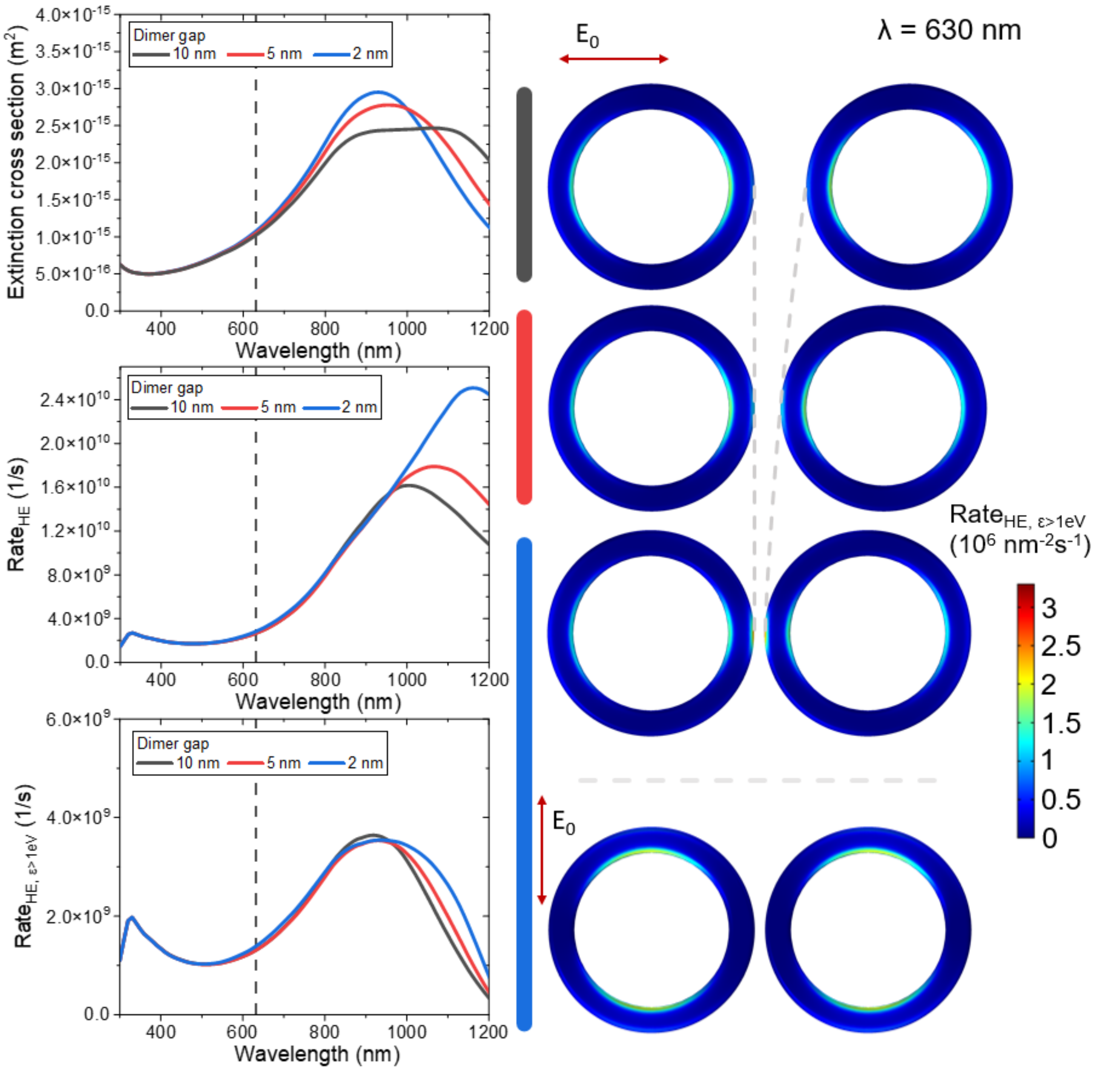

Figure S14. Computational sampling of the effect of the interparticle distance in terms of mode hybridization and interparticle hot spot enhancement for the HNP dimer system. (Left) Spectra obtained by the dimers with three interparticle distances, under unpolarized, normally incident light. From top to bottom, extinction cross sections, excitation rates for intraband hot carriers, without and with a minimum excess energy of 1 eV. (Right) Surface maps for the local excitation rates of intraband hot carriers with energies larger than 1 eV above the Fermi energy of the metal. The top three diagrams show response maps under longitudinal polarization for decreasing gap distances. The bottom panel shows the response map under transverse polarization for the smallest gap. The color bars on the left complement the legend found in the panels on the left.

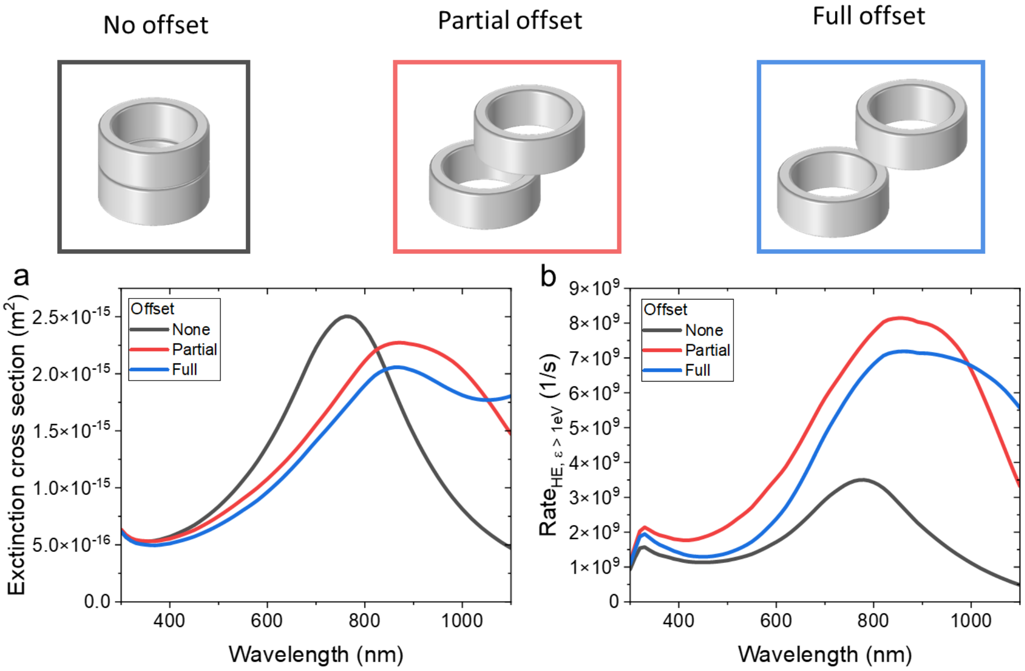

Figure S15. Computational sampling of the effect of horizontal offset in stacked HNPs. (Top) Models corresponding to offsets of 0 nm, 20 nm and 35 nm, respectively. (Bottom) Results of computational simulation of the three models above. (a) Extinction cross sections. (b) Rates of excitation of high-energy intraband hot carriers. The model with partial offset makes a better use of the interparticle hot spots and excites high-energy electrons more rapidly than the others. Of course, in a system with more HNPs, both partially and fully offset top HNP will partially overlap with neighboring bottom HNPs, largely increasing the total contribution to high-energy carrier excitation.

References

[1] Li, J. M.; Liu, J. Y.; Yang, Y.; Qin, D. Bifunctional Ag@Pd-Ag Nanocubes for Highly Sensitive Monitoring of Catalytic Reactions by Surface-Enhanced Raman Spectroscopy. *J. Am. Chem. Soc.* **2015**, *137*, 7039-7042.

[2] Gaussian 16, Revision C.01, Frisch, M. J.; Trucks, G. W.; Schlegel, H. B.; Scuseria, G. E.; Robb, M. A.; Cheeseman, J. R.; Scalmani, G.; Barone, V.; Petersson, G. A.; Nakatsuji, H.; Li, X.; Caricato, M.; Marenich, A. V.; Bloino, J.; Janesko, B. G.; Gomperts, R.; Mennucci, B.; Hratchian, H. P.; Ortiz, J. V.; Izmaylov, A. F.; Sonnenberg, J. L.; Williams-Young, D.; Ding, F.; Lipparini, F.; Egidi, F.; Goings, J.; Peng, B.; Petrone, A.; Henderson, T.; Ranasinghe, D.; Zakrzewski, V. G.; Gao, J.; Rega, N.; Zheng, G.; Liang, W.; Hada, M.; Ehara, M.; Toyota, K.; Fukuda, R.; Hasegawa, J.; Ishida, M.; Nakajima, T.; Honda, Y.; Kitao, O.; Nakai, H.; Vreven, T.; Throssell, K.; Montgomery, J. A., Jr.; Peralta, J. E.; Ogliaro, F.; Bearpark, M. J.; Heyd, J. J.; Brothers, E. N.; Kudin, K. N.; Staroverov, V. N.; Keith, T. A.; Kobayashi, R.; Normand, J.; Raghavachari, K.; Rendell, A. P.; Burant, J. C.; Iyengar, S. S.; Tomasi, J.; Cossi, M.; Millam, J. M.; Klene, M.; Adamo, C.; Cammi, R.; Ochterski, J. W.; Martin, R. L.; Morokuma, K.; Farkas, O.; Foresman, J. B.; Fox, D. J. Gaussian, Inc., Wallingford CT, 2016.

[3] Jamróz, M. H. Vibrational Energy Distribution Analysis (VEDA): Scopes and limitations. *Spectrochimica Acta Part A: Molecular and Biomolecular Spectroscopy* **2013**, *114*, 220-230.

[4] Ünal, Y.; Nassif, W.; Ózaydin, B. C.; Sayin, K. Scale factor database for the vibration frequencies calculated in M06-2X, one of the DFT methods. *Vibrational Spectroscopy* **2021**, *112*, 103189
